# The Mitogen-Activated Protein Kinase Slt2 Promotes Asymmetric Cell Cycle Arrest and Reduces TORC1-Sch9 Signaling in Yeast Lacking the Protein Phosphatase Ptc1

**DOI:** 10.1128/spectrum.05249-22

**Published:** 2023-04-12

**Authors:** Gema González-Rubio, Humberto Martín, María Molina

**Affiliations:** a Departamento de Microbiología y Parasitología. Facultad de Farmacia. Instituto Ramón y Cajal de Investigaciones Sanitarias, Universidad Complutense de Madrid, Madrid, Spain; Université Côte d’Azur, CNRS, Inserm

**Keywords:** *Saccharomyces cerevisiae*, Ptc1, Slt2, MAPK, protein phosphatase, TORC1, Sch9, septin, oxidative stress, autophagy, cell cycle arrest, cell separation, Atg9, RAM pathway, Ace2, Cts1, budding yeast, morphogenesis

## Abstract

Mitogen-activated protein kinase (MAPK) pathways regulate essential processes in eukaryotes. However, since uncontrolled activation of these cascades has deleterious effects, precise negative regulation of signaling flow through them, mainly executed by protein phosphatases, is crucial. Previous studies showed that the absence of Ptc1 protein phosphatase results in the upregulation of the MAPK of the cell wall integrity (CWI) pathway, Slt2, and numerous functional defects in Saccharomyces cerevisiae, including a failure to undergo cell separation under heat stress. In this study, we demonstrate that multibudded *ptc1*Δ cells also exhibit impaired mitochondrial inheritance and that excessive Slt2 kinase activity is responsible for their growth deficiency and daughter-specific G_1_ cell cycle arrest, as well as other physiological alterations, namely, mitochondrial hyperpolarization and reactive oxygen species (ROS) accumulation. We bring to light the fact that sustained Slt2 kinase activity inhibits signaling through the Sch9 branch of the TORC1 pathway in *ptc1*Δ cells, leading to increased autophagy. After cytokinesis, septin rings asymmetrically disassembled in *ptc1*Δ multibudded cells, abnormally remaining at the daughter cell side and eventually relocalizing at the daughter cell periphery, where they occasionally colocalized with the autophagic protein Atg9. Finally, we show that the inability of *ptc1*Δ cells to undergo cell separation is not due to a failure in the regulation of Ace2 and morphogenesis (RAM) pathway, since the transcription factor Ace2 correctly enters the daughter cell nuclei. However, the Ace2-regulated endochitinase Cts1 did not localize to the septum, preventing the proper degradation of this structure.

**IMPORTANCE** This study provides further evidence that the cell cycle is regulated by complex signaling networks whose purpose is to guarantee a robust response to environmental threats. Using the S. cerevisiae eukaryotic model, we show that, under the stress conditions that activate the CWI MAPK pathway, the absence of the protein phosphatase Ptc1 renders Slt2 hyperactive, leading to numerous physiological alterations, including perturbed mitochondrial inheritance, oxidative stress, changes in septin dynamics, increased autophagy, TORC1-Sch9 inhibition, and ultimately cell cycle arrest and the failure of daughter cells to separate, likely due to the absence of key degradative enzymes at the septum. These results imply novel roles for the CWI pathway and unravel new cell cycle-regulatory controls that operate beyond the RAM pathway, arresting buds in G_1_ without compromising further division rounds in the mother cell.

## INTRODUCTION

The cell cycle is an essential process for life by which a progenitor cell gives rise to two identical daughter cells. However, there are multiple examples of asymmetric division, like that carried out by the yeast Saccharomyces cerevisiae, due to its characteristic pattern of duplication by budding, as a result of which mother and daughter cells display several differential features (1). Uncorrected mistakes during cell division result in increased sensitivity to environmental changes, segregation errors of chromosomes and organelles, or even cell death. For this reason, progression along the cell cycle is tightly controlled by checkpoint mechanisms that detect failures and transiently arrest the cell cycle, allowing time for problem-solving (2). The eukaryotic cell division cycle consists of an ordered sequence of four phases called gap 1 (G_1_), DNA synthesis (S), gap 2 (G_2_), and mitosis (M) (3). After chromosome segregation, cytoplasm separation (cytokinesis) by actomyosin ring constriction completes cell division in animal cells. However, in fungi, which have a cell wall, actomyosin ring constriction is coordinated with septum deposition at the division site (4), followed by an additional step consisting of the partial degradation of this septum by hydrolytic enzymes, which is needed for cell separation to occur. In the budding yeast S. cerevisiae, cell separation is asymmetric, since the hydrolases involved in the degradation of the septum are regulated by a transcription factor that accumulates specifically in the daughter nucleus, called Ace2 (5). Nuclear import of Ace2 in late M or early G_1_ triggers the expression of a subset of genes (6), including those encoding the endochitinase Cts1, which is the main enzyme involved in cell separation by the degradation of the chitin-based primary septum (7), and several known or likely glucanases such as Scw11, Dse2, and Dse4, which reinforce the action of Cts1 (8, 9). In addition, Ace2 induces other genes, namely, *DSE1*, *DSE3*, *CST13*, and *PRY3*, whose function has been less well studied. In S. cerevisiae, certain spatial and temporal aspects of the cell cycle are regulated by septins, which are a family of eukaryotic GTP-binding proteins that constitute supramolecular structures that undergo constant remodeling during the cell cycle. At the beginning of the cell cycle, septins accumulate in the presumptive bud site as a patch, which rapidly resolves into a ring that later expands to an hourglass-shaped collar at the bud neck. Before cytokinesis, it splits into a double ring, which is reabsorbed after cell separation (10, 11).

Mitogen-activated protein kinase (MAPK) pathways are highly conserved signal transduction modules that are involved in the generation of cellular responses to different stimuli, playing a crucial role in many aspects of the eukaryotic cell physiology. A canonical MAPK module is composed of three protein kinases that are sequentially activated by phosphorylation, namely, MAPKKK, MAPKK, and MAPK. Once activated, MAPKs phosphorylate numerous substrates to regulate a wide range of functions, including gene expression, metabolism, cell morphology, and cell cycle progression. In S. cerevisiae haploid cells, there are four MAPK pathways: mating, invasive growth, high-osmolarity glycerol (HOG), and cell wall integrity (CWI). The MAPKs involved in these pathways are Fus3, Kss1, Hog1, and Slt2, respectively (12). Although the CWI module contains two MAPKKs, Slt2 is primarily phosphorylated by Mkk1 rather than Mkk2 (13). Phosphorylation of Slt2 leads to the activation of the transcription factors Rlm1 and SBF (Swi4/Swi6) and to the subsequent transcriptional induction of stress-responsive genes (14). Despite being traditionally linked to cell wall remodeling in response to cell wall stress, the CWI pathway is involved in the cellular response to many other stressful conditions (15). Furthermore, the continued discovery of new substrates for Slt2 (16) provides evidence that this pathway plays important roles in the regulation of other cellular processes ranging from mitophagy and pexophagy (17) to cell cycle progression (18).

The duration and magnitude of MAPK activation are crucial for determining a physiological outcome in cells. Among the molecular mechanisms that negatively regulate the signaling flow through the pathway, dephosphorylation of the MAPK cascade components by protein phosphatases is possibly the most important (19). Yeast Ptc1 is a type 2C serine/threonine protein phosphatase that has been involved in many cellular processes that are not shared by other members of its family (20). Ptc1 negatively regulates the HOG (21) and CWI pathways (22) by dephosphorylating the MAPK Hog1 (23) and the MAPKK Mkk1 (24), respectively. Ptc1 also seems to modulate signaling through the mating pathway, although reports differ on whether this regulation is negative (25) or positive (26, 27). The relevance of Ptc1 is evidenced by the numerous functional defects exhibited by yeast cells lacking Ptc1. Many of them, including increased sensitivity to diverse cations, vacuolar fragmentation, alterations in endoplasmic reticulum (ER), and mitochondrial inheritance, as well as defects in growth and cell separation, have been related to the activation of the CWI pathway, since the lack of different components of this pathway substantially alleviates these defects (22, 24, 28, 29). However, the involvement of Slt2 kinase activity in these defects remains to be demonstrated. The absence of Ptc1 has also been associated with other physiological consequences, such as an altered function of the target of rapamycin 1 (TORC1) complex (30), which has not been attributed to deregulation of CWI or any other MAPK signaling pathway. TORC1 coordinates nutrient availability with cell proliferation, promoting anabolic processes like protein translation and ribosome biogenesis, and inhibiting catabolic processes, including autophagy (31). Although the involvement of Slt2 in regulating TORC1 activity upon ER stress has been recently documented (32), the mechanisms underlying this regulation are still unclear.

In this work, we significantly extended our understanding of the impact of the absence of Ptc1 on yeast physiology and the role of the CWI pathway in this process.

## RESULTS

### Ptc1 is necessary for cell separation and cell cycle progression of daughter cells under distinct CWI pathway-activating conditions.

**FIG 1:**
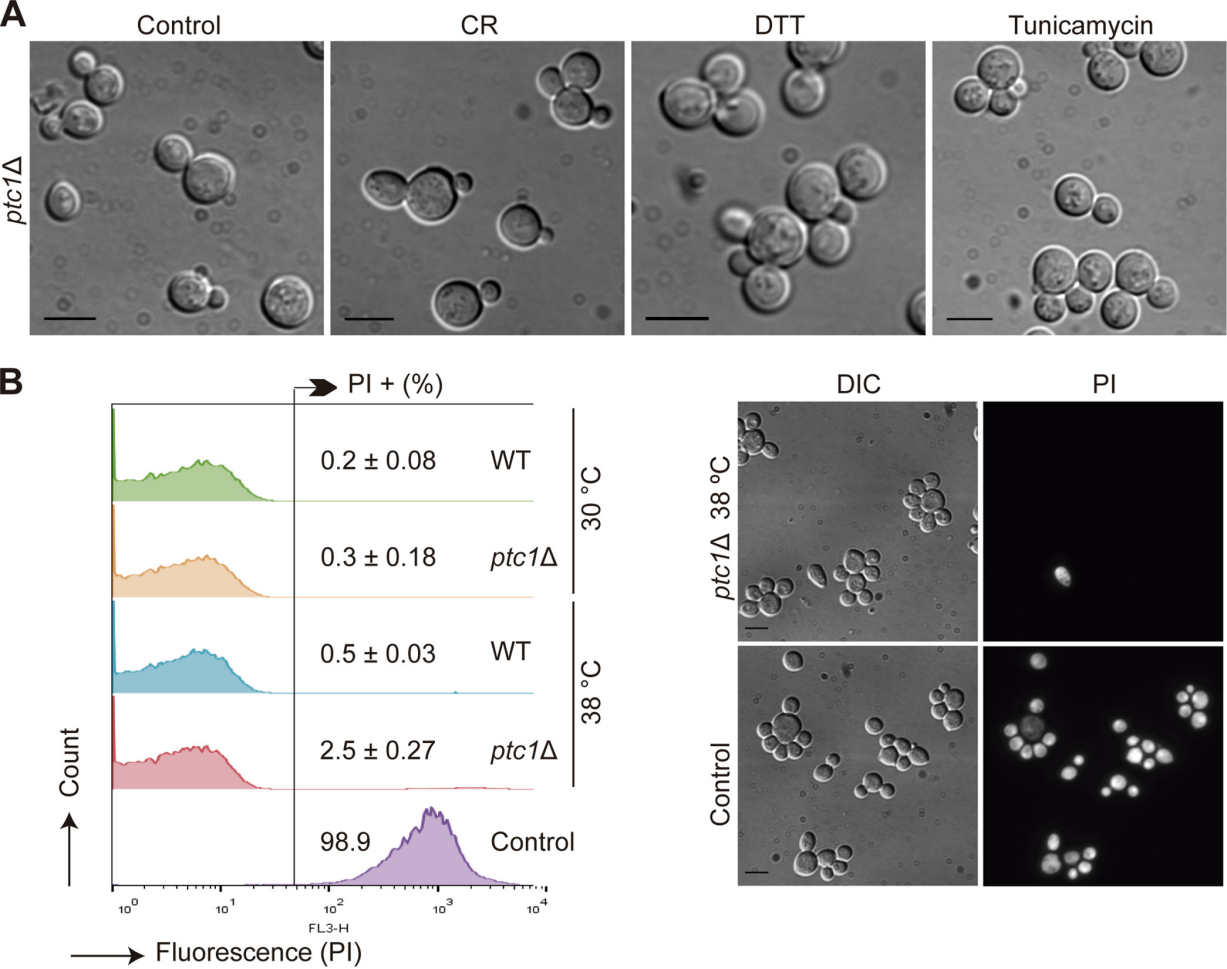
Effects of CWI-activating compounds on the phenotype and cell death of the *ptc1*Δ mutant. (A) Cultures of the YSTH14 (*ptc1*Δ) strain growing in YPD to exponential phase were cultured for an additional 7 h at 25°C in the absence of stress (control) or in the presence of 5 μg/mL CR, 2 mM DTT, or 1 μg/mL tunicamycin, as indicated, and observed by DIC microscopy. (B) Stacked histograms (*n* = 20,000) showing PI fluorescent signal by FCM analysis (left) and DIC and fluorescence microscopy images (right) of exponentially growing cultures of the strains Y3656 (WT) and YSTH14 (*ptc1*Δ) cultured for an additional 12 h at 30°C or 38°C and stained with PI. Controls consisted of heat-treated dead cells. Bars = 5 μm.

Previous studies have shown that cells lacking Ptc1 exhibit two high-temperature-dependent cell cycle defects: a failure to undergo cell separation and an apparent asymmetric loss of viability in daughter cells. These alterations lead to the accumulation of multibudded cells (24, 28, 33). We first investigated whether such phenotypic abnormalities were also induced by insults other than heat stress that trigger the activation of the CWI pathway by different means, such as Congo red (CR), which specifically alters the yeast cell wall, or dithiothreitol (DTT) and tunicamycin, which increase the load of unfolded proteins within the ER (15). As shown in Fig. 1A, when cells were stimulated with CR, DTT, or tunicamycin, *PTC1* deletion resulted in the appearance of multibudded cells, indicating that Ptc1 is necessary for cell separation under different conditions that turn on the yeast CWI pathway and suggesting that CWI hyperactivation is responsible for the cell cycle alterations shown by cells lacking Ptc1.

We next investigated whether the daughter cells that accumulated in the *ptc1*Δ mutant at 38°C were viable. To address this question, we treated cells with propidium iodide (PI), which stains nonviable cells that have lost membrane permeability. PI staining revealed that only 2.5% of *ptc1*Δ cells at 38°C were permeable to the dye (Fig. 1B). These results suggest that daughter cells of *ptc1*Δ mutants subjected to heat stress undergo a cell cycle arrest that does not lead to loss of viability.

### Multibudded cells can be readily quantified by flow cytometry.

**FIG 2:**
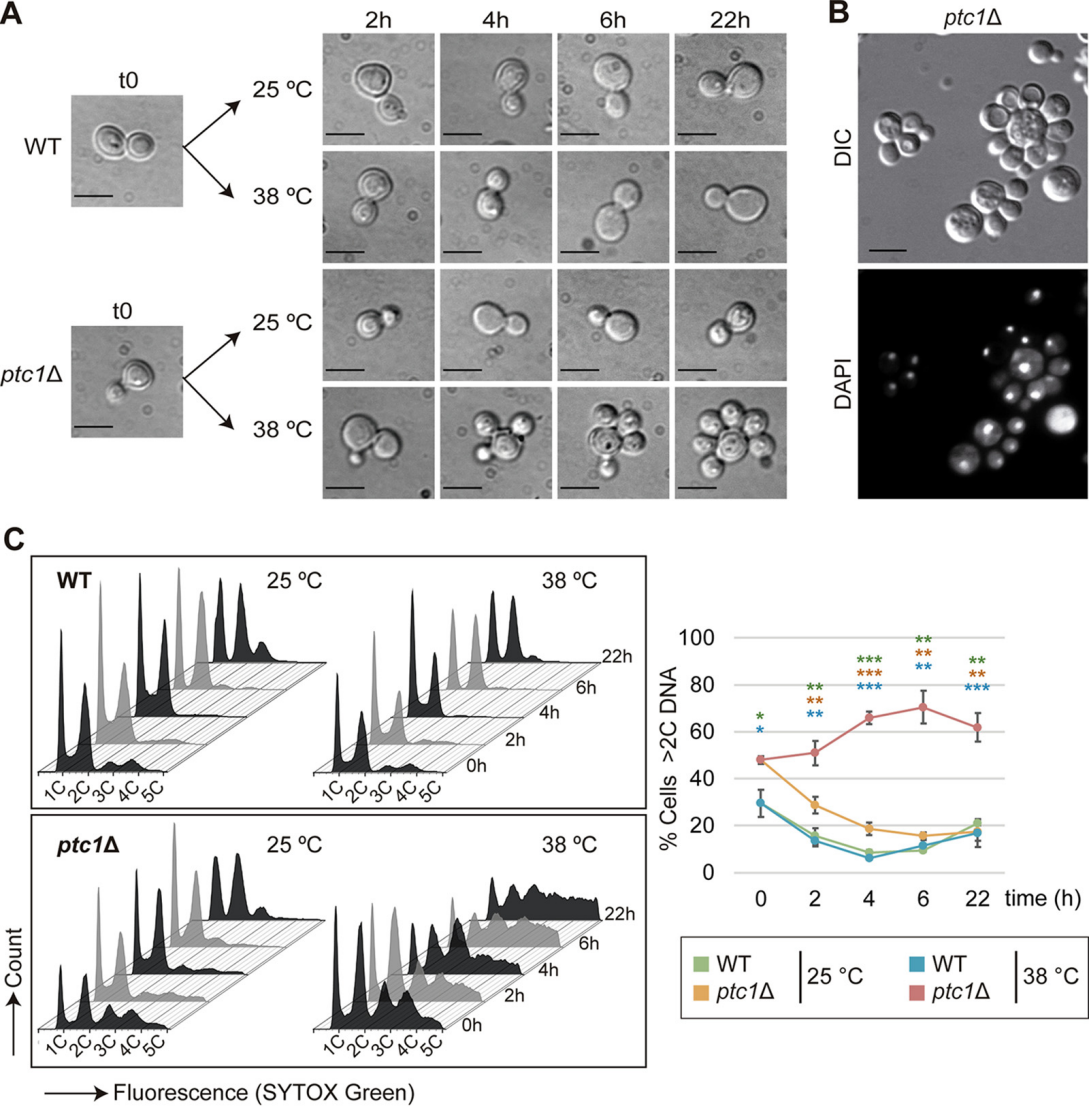
Dynamics of the multibudding phenotype. (A) DIC microscopy images of exponentially growing cultures of the strains Y3656 (WT) and YSTH14 (*ptc1*Δ) incubated for 22 h at 25 or 38°C. Bar = 5 μm. (B) DIC and fluorescence microscopy images of exponentially growing cells of the YSTH14 (*ptc1*Δ) strain incubated at 38°C for 18 h and stained with DAPI. Bar = 5 μm. (C) Fluorescence histogram (left) and line graph (right) showing the DNA content (1C, 2C, 3C, 4C, or 5C) and percentage of cells with more than double DNA content (>2C DNA), respectively, of the same cell cultures as in panel A, stained at the indicated times with Sytox green and analyzed by FCM (*n* = 30,000). Error bars indicate the standard deviation (SD) for three independent experiments. Student’s *t* test was used to compare the percentage of cells with a DNA content higher than two copies (>2C DNA) between the *ptc1*Δ strain at 38°C and the WT at 25°C (green), the *ptc1*Δ strain at 25°C (orange), or the WT at 38°C (blue). *, *P* < 0.05; **, *P* < 0.01; ***, *P* < 0.001.

The accumulation of successive buds in heat-stressed *ptc1*Δ cells can be easily visualized by differential interference contrast (DIC) microscopy (Fig. 2A). By DAPI (4′,6-diamidino-2-phenylindole) nuclear staining, we demonstrated that, in multibudded cells, the mother and all the daughters contained their own nucleus (Fig. 2B), indicating that nuclear division and inheritance are successfully completed in multibudded *ptc1*Δ cells. These results prompted us to follow the dynamics of the multibudding phenotype by flow cytometry (FCM) analysis. We used Sytox green, a fluorescent dye that quantitatively binds to DNA and emits fluorescence with an intensity corresponding to the cellular DNA content (34). Fluorescence histograms of asynchronous cell populations stained with Sytox green accurately showed that, when grown at 38°C, the *ptc1*Δ mutant exhibited an increase in the population of cells with 3, 4, and 5 copies of DNA (3C, 4C, and 5C) over time (Fig. 2C, left), which perfectly matched the characteristic successive bud accumulation displayed by cells lacking Ptc1. The highest proportion of multibudded cells (>2C DNA) was reached at 6 h (Fig. 2C, right). These results are evidence that cytometric analysis of DNA content with Sytox green constitutes a fast and reliable tool for quantifying the proportion of multibudded cells. They also suggest that the defects in growth and cell separation displayed by *ptc1*Δ mutants at 38°C are not transitory, due to a cell cycle delay, but result from a daughter-specific blockage of cell cycle progression at G_1_.

### Under heat stress, the lack of Ptc1 causes an increase in the phosphorylation levels of Slt2 but not of other MAPKs.

**FIG 3:**
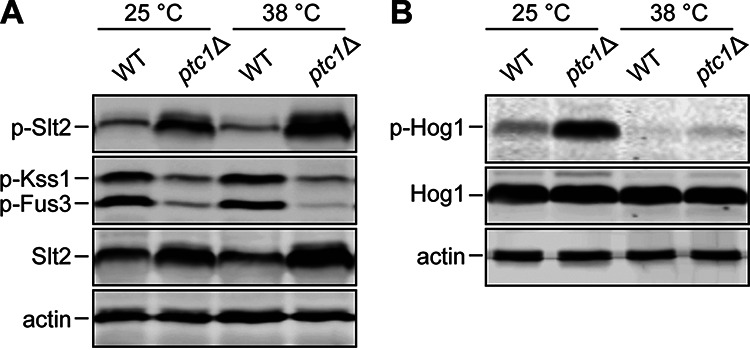
Effect of *PTC1* deletion on MAPK phosphorylation. (A and B) Exponentially growing cultures of Y3656 (WT) and YSTH14 (*ptc1*Δ) strains were incubated for an additional 6 h at 25°C or 38°C. Cell extracts were analyzed by immunoblotting with anti-phospho-p44/42 MAPK and anti-Mpk1, which detect dually phosphorylated Slt2, Kss1, and Fus3 and Slt2, respectively (A); anti-phospho-p38 MAPK and anti-Hog1, which detect dually phosphorylated Hog1 and Hog1, respectively (B); and anti-actin as a loading control (A and B). In each case, a representative blot from three independent experiments is shown.

In addition to the CWI pathway, other MAPK cascades have been identified as regulated by Ptc1, namely, the HOG and mating pathways (20). Specifically, *PTC1* deletion substantially prevents shmooing and reduces activation of Fus3 (26), leads to a significant delay in Hog1 activation and inactivation (23), and results in hyperphosphorylation of Slt2 (22). Since any of these MAPKs could contribute to the phenotypes shown by the *ptc1*Δ mutant at high temperatures, we analyzed in parallel, by Western blotting with different anti-p-MAPKs antibodies, the phosphorylation state of Slt2, Kss1, Fus3, and Hog1 in cells lacking Ptc1 subjected to heat stress. Slt2 hyperphosphorylation in *ptc1*Δ cells was observed at both 25°C and 38°C, with this effect being higher upon heat stress. Moreover, increased levels of phosphorylated Slt2 correlated with a larger amount of this MAPK (Fig. 3A). Given that Slt2 activity regulates its own transcription (35), we conclude that in *ptc1*Δ mutants, Slt2 is both hyperphosphorylated and hyperactive (Fig. 3A).

In contrast, *PTC1* deletion led to a reduction in the phospho-Fus3 amount, which was more evident at 38°C (Fig. 3A). We found that the phosphorylation levels of Kss1, the MAPK involved in the mating and in the invasive filamentous pathway, also decreased in *ptc1*Δ cells similarly at 25 and 38°C (Fig. 3A).

Finally, *PTC1* deletion led to an increase in Hog1 phosphorylation level at 25°C. Intriguingly, when cells were grown at 38°C, Hog1 was not hyperphosphorylated (Fig. 3B). These results rule out the possibility that the phenotypes shown by *ptc1*Δ cells under heat stress result from hyperphosphorylation of Kss1, Fus3, or Hog1, suggesting that Slt2 hyperphosphorylation is responsible for the phenotypic defects exhibited by *ptc1*Δ mutants at 38°C.

### Deletion of *MKK1* or *RLM1*, but not of *SWI4* or *SWI6*, reduces the defects in cell separation and growth of *ptc1*Δ mutant cells.

Attenuation of signaling by removal of certain components of the CWI pathway ameliorates many of the phenotypes derived from the absence of Ptc1. Hence, the deletion of *MKK1*, the main MAPKK involved in dual phosphorylation and activation of Slt2 (13), increases the tolerance of cells lacking Ptc1 to cell wall-altering compounds (24). Moreover, it reduces the vacuolar fragmentation, alterations in the budding pattern, and cell separation defects shown by the *ptc1*Δ mutant at 37°C (24). In the same way, mutation of the Slt2-activated transcription factor Rlm1 (36) reduced the sensitivity to CR and calcofluor white (CFW) of a *ptc1*Δ strain (28), suggesting that hyperactivation of Slt2 is involved in these phenotypic defects (24).

**FIG 4:**
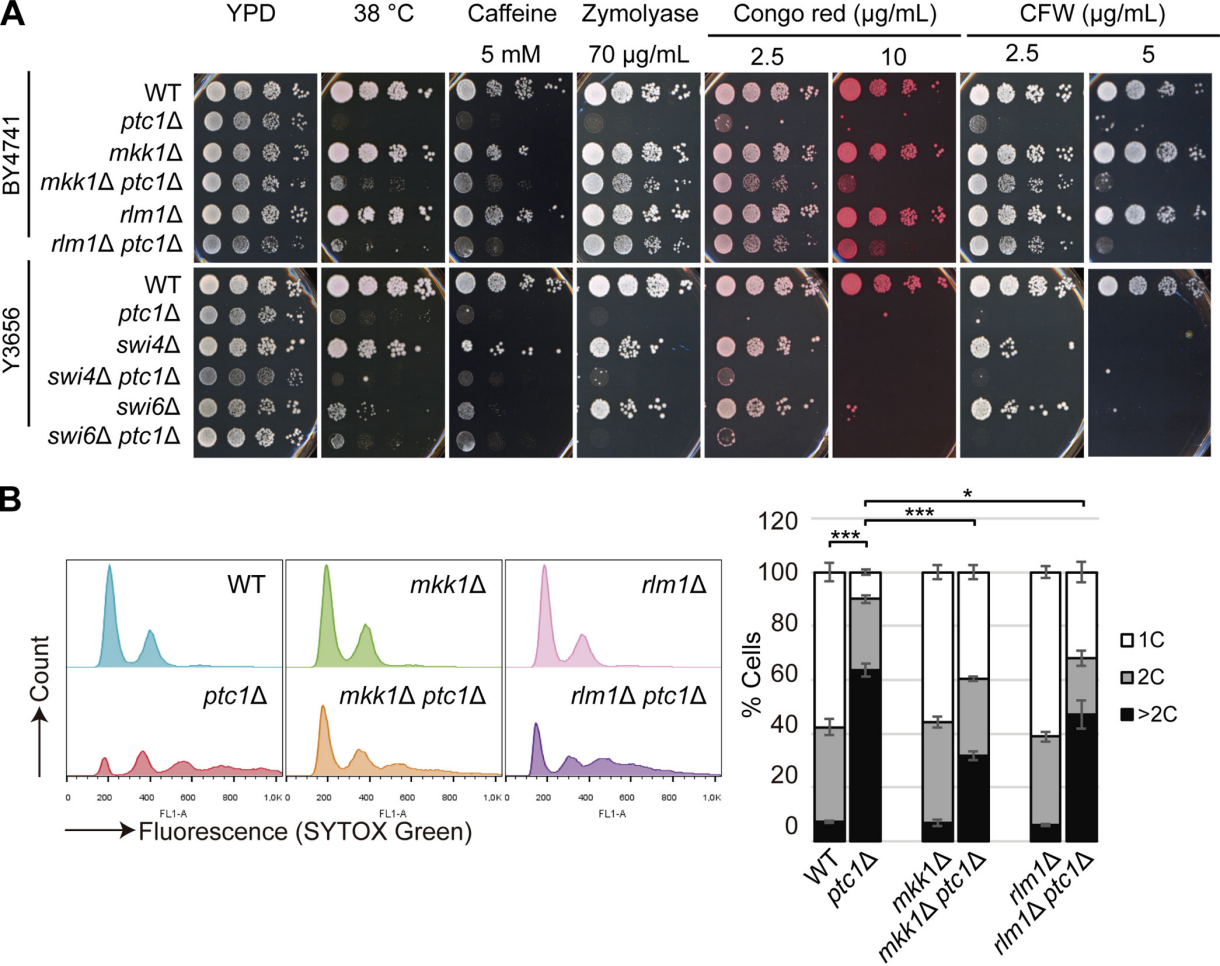
Comparative analysis of the effects of deletion of *MKK1*, *RLM1*, *SWI4*, or *SWI6* in the *ptc1*Δ background. (A) Growth assay of WT and the indicated mutants under specified conditions. Photographs were taken after 72 h. (B) Stacked histograms (*n* = 15,000) showing Sytox green fluorescent signal by flow cytometry (left) and graph showing the percentage of populations with 1C, 2C, and >2C DNA copies (right) of the strains in panel A (top). Exponentially growing cells were incubated 6 h at 38°C, stained with Sytox green, and analyzed by FCM. Error bars indicate the SD for three independent experiments. *, *P* < 0.05; ***, *P* < 0.001 (Student’s *t* test).

Once activated, Slt2 leads to the activation not only of Rlm1 but also of the transcription factor SBF (composed of Swi4 and Swi6 proteins) (37). To further investigate the downstream components of Slt2 that are involved in *ptc1*Δ phenotypic traits, we first performed a comparative growth assay, including yeast strains lacking Mkk1, Rlm1, Swi4, or Swi6. As expected from previous reports, in both genetic backgrounds analyzed, *ptc1*Δ cells showed a high sensitivity to different CWI-activating compounds (Fig. 4A). Both *MKK1* and *RLM1* deletions increased the tolerance of the *ptc1*Δ mutant to high temperature, caffeine, Zymolyase, CR, and CFW. However, the absence of *SWI4* or *SWI6* only very slightly improved the growth of *ptc1*Δ cells in the presence of low concentrations of CR (Fig. 4A), suggesting that the SBF-mediated response is not crucial to the hypersensitivity to cell wall-altering compounds shown by cells lacking Ptc1.

Next, we comparatively analyzed the effect of *MKK1* and *RLM1* deletion on cell separation defects shown by *ptc1*Δ mutants at 38°C. FCM analysis using Sytox green revealed that, although the absence of both Mkk1 and Rlm1 led to a reduction in the proportion of *ptc1*Δ cells with >2C DNA content, this effect was higher in *mkk1*Δ *ptc1*Δ than *rlm1*Δ *ptc1*Δ cells (Fig. 4B). Taken together, these results provide evidence that the growth and cell separation defects shown by *ptc1*Δ cells subjected to heat stress are only partially due to the Rlm1-mediated response and that other Slt2 substrates, different from the transcription factors Rlm1 and SBF (Swi4/Swi6), contribute to this multibudding phenotype.

### Slt2 kinase activity is required for the defects in growth and cell separation shown by cells lacking Ptc1.

**FIG 5:**
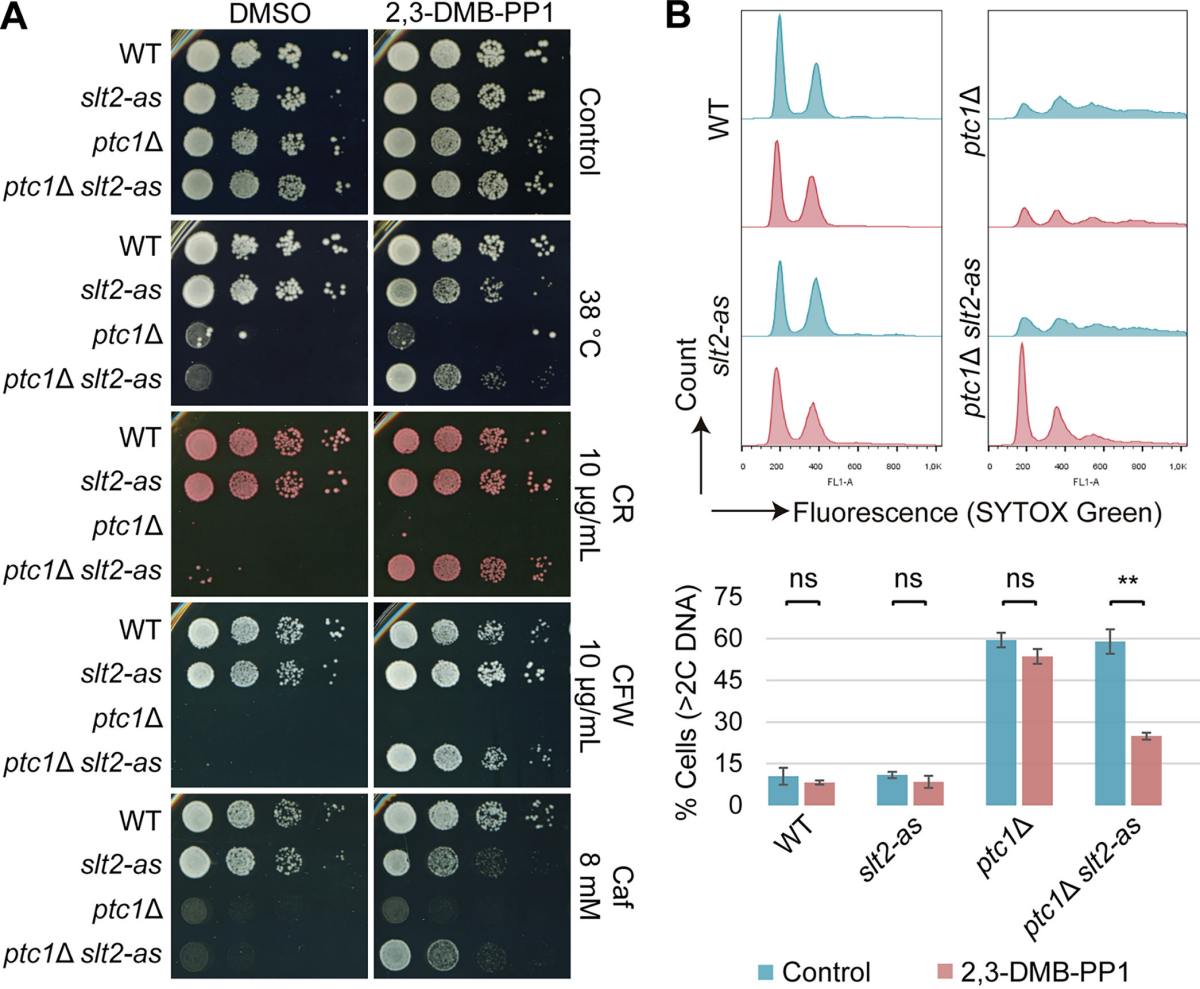
Effect of Slt2 inhibition on sensitivity and cell separation defects of *ptc1*Δ cells under CWI-activating conditions. (A) Growth assay of WT and the indicated mutants under the indicated conditions, in the absence (DMSO) or presence of 5 μM 2,3-DMB-PP1. Photographs were taken at 72 h, except for the caffeine plates (120 h). (B) Stacked histograms (*n* = 26,000) of Sytox green fluorescent signal (top) and graph showing the percentage of populations with >2C DNA copies (bottom) of the strains used for panel A. Exponentially growing cultures treated with 5 μM 2,3-DMB-PP1 or not (control) were incubated for 6 h at 38°C, stained with Sytox green, and analyzed by FCM. Error bars indicate the SD for three independent experiments. **, *P* < 0.01; ns, not statistically significant (*P* > 0.05) (Student’s *t* test).

Deletion of *SLT2* did not recover growth in a *ptc1*Δ mutant due to the need for the CWI pathway response to survive under cell wall stress (28). Therefore, to demonstrate whether Slt2 kinase activity is the basis of the defects in cell separation and cell cycle progression of multibudded *ptc1*Δ cells, we used an analog-sensitive mutant of Slt2 (Slt2-as) that can be specifically inhibited in a dose-dependent manner by bulky kinase inhibitor analogs, such as 4-amino-1-tert-butyl-3-(2,3-dimethylbenzyl)pyrazolo[3,4-d]pyrimidine (2,3-DMB-PP1) (38). To this end, we generated a *ptc1*Δ strain carrying Slt2-as as the unique Slt2 version and analyzed its sensitivity to different CWI pathway-activating compounds and the percentage of multibudded cells (>2C DNA) growing at 38°C in the presence of 2,3-DMB-PP1. The growth assay of yeast cells revealed that the inhibition of Slt2-as kinase activity markedly improved the tolerance of *ptc1*Δ mutant to high temperature, CR, CFW, and caffeine (Fig. 5A). FCM data on DNA content showed that both wild-type (WT) and Slt2-as strains exhibited two peaks (1C and 2C) regardless of the presence or absence of the kinase inhibitor (Fig. 5B, top). The addition of 2,3-DMB-PP1 did not alter the DNA copy number of *ptc1*Δ cells, which exhibited up to five peaks (Fig. 5B, top). However, when added to the *ptc1*Δ *slt2*-as strain, the kinase inhibitor promoted a significant reduction in the percentage of multibudded cells (>2C DNA) (Fig. 5B, bottom). Altogether, these results provide conclusive evidence that both the increased sensitivity and the multibudding phenotype showed by *ptc1*Δ mutants growing under CWI pathway-activating conditions are caused by elevated Slt2 kinase activity.

### Excessive Slt2 kinase activity leads to oxidative stress in heat-stressed *ptc1*Δ cells, which display defects in mitochondrial inheritance.

**FIG 6:**
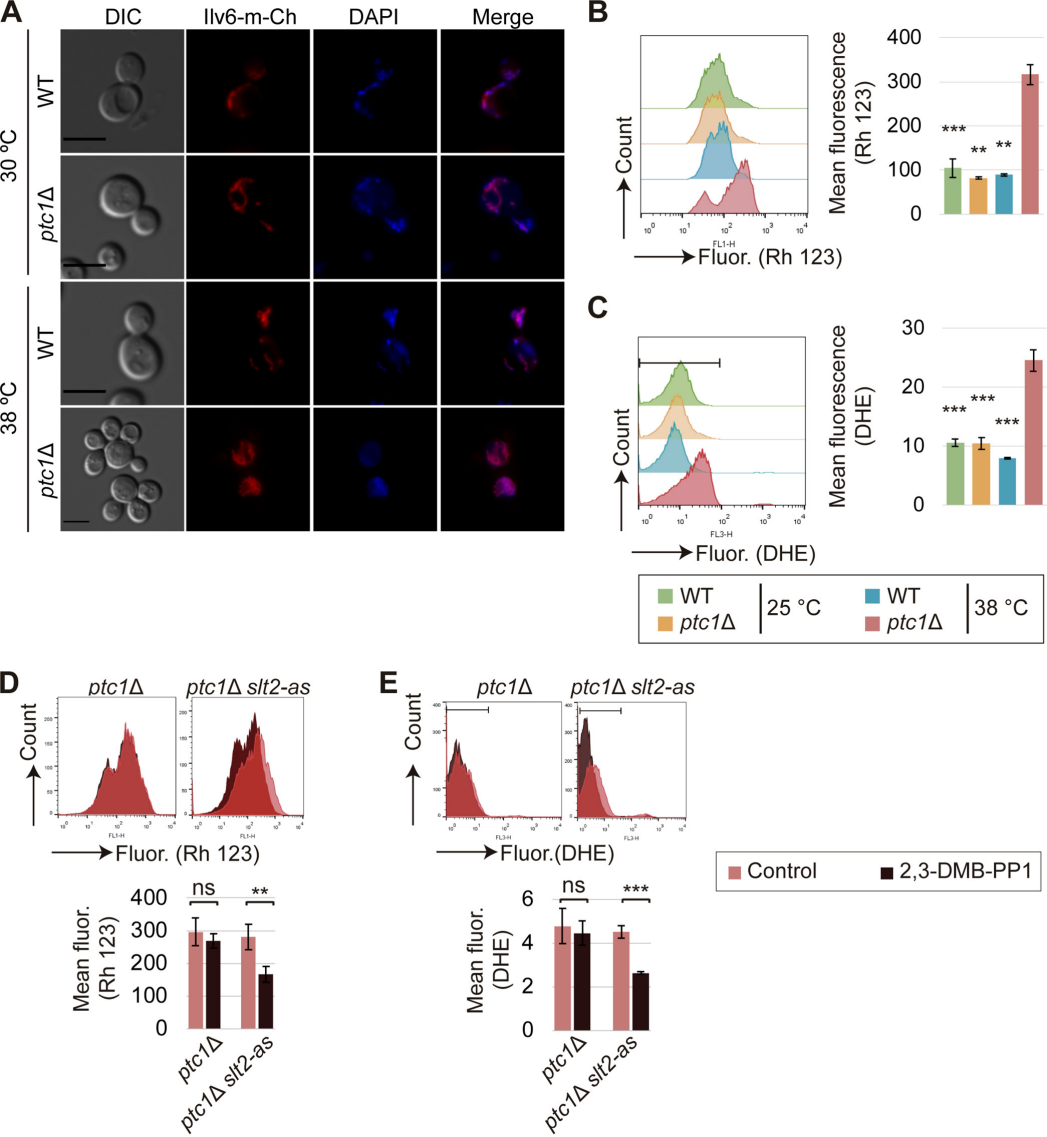
Effect of *PTC1* deletion and Slt2 inhibition on mitochondrial and mtDNA inheritance and oxidative stress. (A) Exponentially growing cultures of Y3656 (WT) and YSTH14 (*ptc1*Δ) strains were transformed with Ilv6-m-Cherry-expressing plasmid pOB08 and incubated at 30 or 38°C for 6 h. Cells were stained with DAPI and visualized by DIC and fluorescence microscopy. Bar = 5 μm. (B and C) Histograms showing Rh 123- or DHE-derived fluorescence and graphs showing the mean Rh 123- or DHE-derived fluorescence of each population. Exponentially growing cultures of Y3656 (WT) and YSTH14 (*ptc1*Δ) strains were incubated for 12 h at 30°C or 38°C. Cells were stained with Rh 123 or with DHE, as indicated, and analyzed by FCM (*n* = 20,000). (D and E) Exponentially growing cultures of YSTH14 (*ptc1*Δ) and YGGR19 (*ptc1*Δ *slt2*-as) strains were incubated in the absence (control) or presence of 5 μM 2,3-DMB-PP1 for 12 h at 38°C. Cells were stained with Rh 123 or with DHE, as indicated, and analyzed by FCM (*n* = 11,000). Error bars indicate the SD for three independent experiments. **, *P* < 0.01; ***, *P* < 0.001; ns, not statistically significant *P* > 0.05 (Student’s *t* test).

Cells lacking Ptc1 exhibit an increased frequency of petite colonies (39). Given that petites have been commonly associated with alterations in mitochondria (40), we first analyzed mitochondrial morphology and inheritance and we found that in *ptc1*Δ cells subjected to heat stress, mitochondria fail to move from the mother cell to the multiple buds so that the presence of both mitochondria and mitochondrial DNA (mtDNA) in daughter cells is negligible (Fig. 6A). In addition, the mitochondria of the mothers are not found to be in tubular form, but rather, they are more spherical and larger than those observed in wild-type or unstressed *ptc1*Δ cells (Fig. 6A). We next assessed mitochondrial respiratory function using rhodamine 123 (Rh 123) and FCM analysis. Rh 123 accumulates in the mitochondria in response to the negative membrane potential. As shown in Fig. 6B, under heat stress, *ptc1*Δ mutants exhibited a significant increase in cellular Rh 123 fluorescence. This rise in mitochondrial potential led to overproduction of reactive oxygen species, as shown by dihydroethidium (DHE) staining (Fig. 6C). DHE enters living cells, where it reacts with reactive oxygen species (ROS), generating ethidium, which emits fluorescence. Hence, the intensity that result from the DHE staining correlates with ROS levels (41). Taken together, these results demonstrate that *PTC1* deletion, combined with heat stress, causes defects in mitochondrial inheritance, morphology, and function.

To determine if the oxidative stress shown by *ptc1*Δ cells at high temperature is a consequence of Slt2 hyperactivation, we analyzed the Rh123 and DHE staining of *ptc1*Δ mutants carrying Slt2-as, cultured in the presence and absence of the kinase inhibitor 2,3-DMB-PP1. 2,3-DMB-PP1 significantly reduced the mitochondrial hyperpolarization and the high level of ROS shown by *ptc1*Δ cells (Fig. 6B). These results provide strong support for the model in which Slt2, through its kinase activity, gives rise to oxidative stress in heat-stressed cells lacking Ptc1.

### Slt2 kinase activity inhibits signaling through the Sch9 branch of the TORC1 pathway in *ptc1*Δ cells.

**FIG 7:**
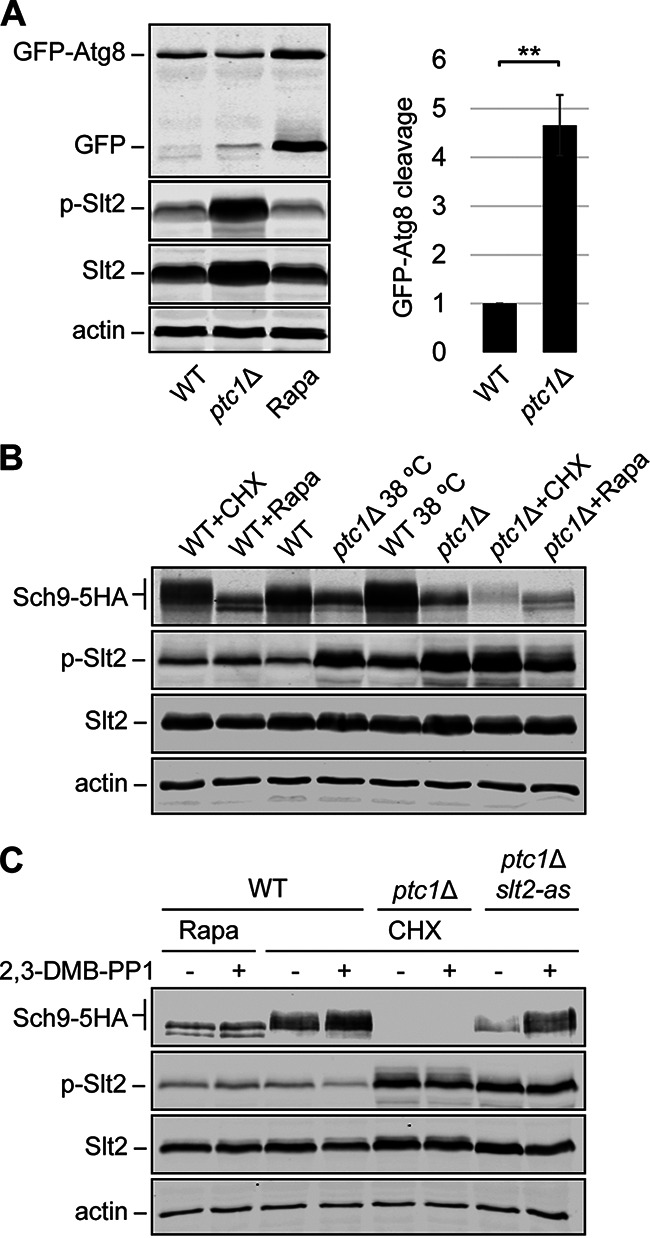
Effect of *PTC1* deletion on autophagic activity and TORC1 activation. (A) Processing of GFP-Atg8 for monitoring autophagy. Exponentially growing cells of Y3656 (WT) and YSTH14 (*ptc1*Δ) strains carrying the plasmid pRS316[GFP-Atg8] were cultured for 6 h at 38°C with rapamycin (Rapa; 0.2 μg/mL) when indicated. Cell extracts were analyzed by immunoblotting with anti-GFP, anti-phospho-p-44/42 MAPK (p-Slt2), anti-Mpk1 (Slt2), and anti-actin (as a loading control). A representative blot from three independent experiments is shown. Bands were quantified using Odyssey software, and GFP-Atg8 cleavage was calculated as the ratio of the free GFP band to the total GFP signal in the lane and then normalized to the WT value. Error bars indicate the SD for three independent experiments. **, *P* < 0.01 by Student’s *t* test. (B and C) Immunoblots showing the phosphorylation of Sch9. Exponentially growing cells of Y3656 (WT), YSTH14 (*ptc1*Δ), and YGGR19 (*ptc1*Δ *slt2*-as) strains carrying the plasmid pRS416 *SCH9-5HA* were treated, when indicated, with cycloheximide (CHX, 25 μg/mL) or 0.2 μg/mL Rapa, in the absence (−) or presence (+) of 5 μM 2,3-DMB-PP1 and incubated for 6 h at 30°C or 38°C, when specified. Cell extracts were analyzed with anti-HA, anti-phospho-p-44/42 MAPK (p-Slt2), anti-Mpk1 (Slt2), and anti-actin (as loading control). A representative blot from three independent experiments is shown.

Oxidative stress causes cell damage by oxidization of proteins, lipids, and DNA, leading to the activation of autophagy (42). To investigate the correlation between oxidative stress and autophagic activity in *ptc1*Δ mutants, we followed autophagic flux by monitoring the vacuolar delivery and subsequent breakdown of the autophagic protein Atg8, N-terminally fused to the green fluorescent protein (GFP). The proteolysis of GFP-Atg8 releases a relatively stable GFP moiety, which accumulates in the vacuole as autophagy proceeds. Accordingly, the increase in free-GFP levels can be detected and quantified by Western blotting and correlated with the autophagic rate (43). As shown in Fig. 7A, *PTC1* deletion resulted in increased GFP-Atg8 cleavage, indicative of enhanced autophagic activity, compared to WT cells.

Several studies have demonstrated that, under favorable growth conditions, TORC1 inhibits autophagy and that inactivation of TORC1 leads to an increase in autophagic activity (44). One of the proteins involved in this regulation is Sch9, a TORC1 substrate that induces autophagy when simultaneously inactivated with protein kinase A (PKA) (45). Sch9 is activated by the phosphorylation of several residues within its C terminus (46). Hence, unphosphorylated and, thus, inactivated Sch9 leads to the induction of autophagy (45). To determine if the increase in autophagic activity shown by *ptc1*Δ mutants could be due to an inactivation of the TORC1-Sch9 pathway, we analyzed the phosphorylation state of Sch9, which can be observed as a mobility shift by SDS-PAGE. To this end, WT and *ptc1*Δ strains carrying a plasmid expressing C-terminally five-hemagglutinin (5HA)-tagged Sch9 were grown with and without heat stress. Given that Sch9 becomes dephosphorylated upon rapamycin treatment and hyperphosphorylated in the presence of cycloheximide (CHX) (46), which inhibits protein synthesis in eukaryotic cells, these compounds were employed as negative and positive controls, respectively, of the phosphorylation state of Sch9. As expected, WT cells treated with rapamycin exhibited nonphosphorylated Sch9, while CHX addition led to the appearance of Sch9-5HA bands of lower mobility, corresponding to the different phosphorylation states of this protein (Fig. 7B, first and second lanes). In line with this, rapamycin also increased autophagic activity, as revealed by GFP-Atg8 cleavage in WT cells (Fig. 7A). Wild-type cells cultured at 38°C exhibited an increase in Sch9 phosphorylating levels in comparison to cells growing at 30°C (Fig. 7B, third and fifth lanes). These results show that the TORC1-Sch9 branch of S. cerevisiae is activated by heat stress. However, cells lacking Ptc1 showed very low Sch9 phosphorylation that did not further increase at 38°C (Fig. 7B, fourth and sixth lanes). It is interesting that the total amount of Sch9 in *ptc1*Δ mutants was lower under all conditions tested than in their counterpart WT cells (Fig. 7B). Moreover, when treated with CHX, Sch9 decreases to almost negligible levels in *ptc1*Δ cells (Fig. 7B, seventh lane). These results provide clear evidence that the absence of Ptc1 causes defects in both Sch9 amount and activation, suggesting the involvement of this phosphatase in the regulation of the TORC1-Sch9 pathway.

Considering that many of the phenotypic alterations shown by *ptc1*Δ mutants are due to high Slt2 kinase activity, we decided to investigate the effect of Slt2-as inhibition on Sch9 stability and activation. To this end, we analyzed, by immunoblotting, extracts of WT, *ptc1*Δ, and *ptc1*Δ *slt2*-as cells carrying the plasmid expressing Sch9-5HA and treated with CHX, with and without the kinase inhibitor 2,3-DMB-PP1. As expected, in WT cells, rapamycin treatment kept Sch9 in a nonphosphorylated state, while the addition of CHX led to the appearance of bands with lower electrophoretic mobility (Fig. 7C, second and fourth lanes, respectively), in both the absence and presence of 2,3-DMB-PP1. When added to *ptc1*Δ cells, CHX caused an almost total disappearance of Sch9, also independently of the presence of the kinase inhibitor (Fig. 7C, fifth and sixth lanes). However, the addition of 2,3-DMB-PP1 to the *ptc1*Δ *slt2*-as strain caused a marked increase in both the amount and phosphorylation of Sch9 (Fig. 7C, eighth lane). Taken together, these results demonstrate that the specific inhibition of Slt2 kinase activity significantly reduces the Sch9 downregulation exhibited by cells lacking Ptc1 and reveals a new role of the CWI MAPK Slt2 as a modulator of the Sch9 branch of the TORC1 pathway.

### Septin rings asymmetrically disassemble and septins relocalize at the daughter cell periphery in multibudded *ptc1*Δ cells.

**FIG 8:**
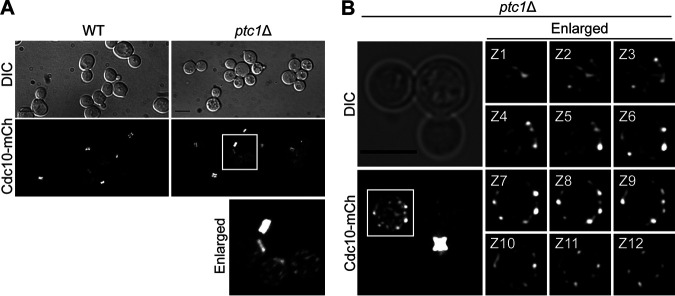
Septin dynamics in multibudded *ptc1*Δ cells. (A) DIC and fluorescence microscopy images of exponentially growing cells of YGGR9 (Cdc10-mCh, WT) and the isogenic mutant YGGR10 (*ptc1*Δ Cdc10-mCh) strains incubated at 38°C for 6 h. (B) Confocal laser microscopy images of 12 Z-sections (enlarged images) and the corresponding Z-stack (lower left) of exponentially growing cells of strain YGGR45 (*ptc1*Δ CDC10-mCh) incubated at 38°C for 6 h. Bars = 5 μm.

Cell cycle progression in eukaryotes is coordinated with dynamic remodeling of the septin-containing structures. In S. cerevisiae, prior to cell division, the hourglass-like septin structure splits into two separate rings, which disappear once cell separation has been properly completed (47). We asked whether cell cycle arrest in buds of multibudded *ptc1*Δ cells correlates with an alteration in septin dynamics. We first examined S. cerevisiae cells that expressed from its native promoter at its own chromosomal locus a derivative of Cdc10 to which the fluorophore mCherry (mCh) had been fused in frame to the C-terminal end. We found that, in *ptc1*Δ mutants, after septin collar splitting, multibudded cells exhibited an asymmetrical disassembly of the two septin rings. While the ring localized in the mother side of the mother-bud neck disappeared before the emergence of the next bud, the septin ring in the daughter side remained assembled throughout successive budding events, although it finally disappeared after several cell division rounds (Fig. 8A). This provides further evidence that in multibudded *ptc1*Δ cells there is an asymmetric cell cycle arrest that affects only the daughters.

Together with the delayed disassembly of the septin ring, we observed that, as septins were reabsorbed, they formed punctiform structures in daughter cells (Fig. 8), which mainly localized at the cell periphery, as revealed by confocal microscopy (Fig. 8B). Thus, while the last-born daughter cell lacked these septin structures, the older the buds were, the more cortical septin puncta they accumulated. This alteration in septin dynamics was observed in 96% of multibudded *ptc1*Δ cells (*n* = 100).

**FIG 9:**
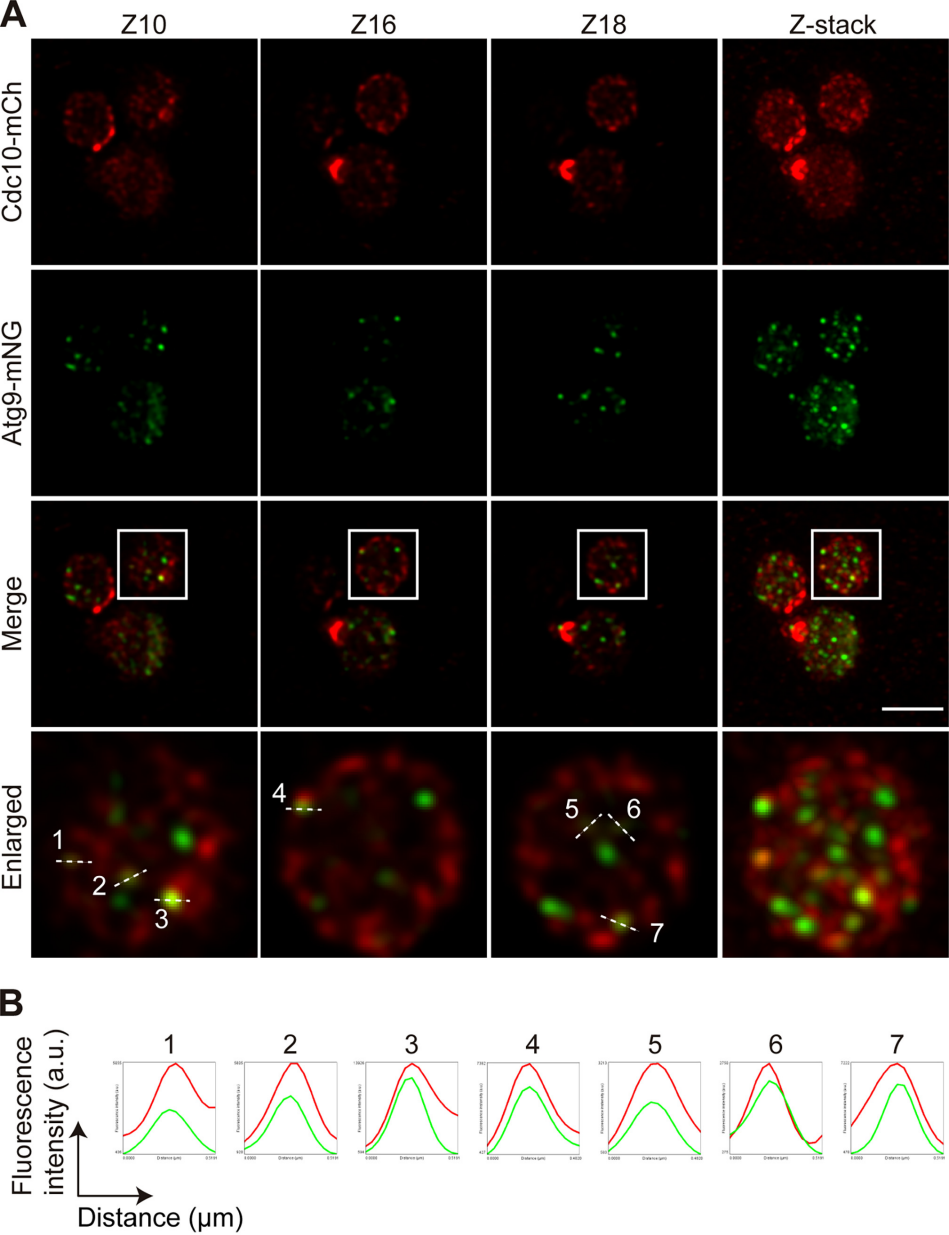
Localization of Atg9 and Cdc10 in multibudded *ptc1*Δ cells. Exponentially growing cells of strain YGGR45 (*ptc1*Δ Atg9-mNG, Cdc10-mCh) were incubated for 6 h at 38°C and analyzed by confocal laser microscopy. (A) Four representative confocal planes and the Z-stack corresponding to a total of 24 Z planes are shown. Bar = 5 μm. (B) Histograms showing arbitrary units (a.u.) of fluorescence intensity versus distance, represented by the dashed lines in the enlarged images in panel A.

Previous work indicated that, upon autophagy induction, septin complexes appear as puncta and sometimes as noncanonical ring-like structures that occasionally colocalize with preautophagosomal structures (PAS) and autophagosomes, suggesting a possible role of these cytoskeleton proteins in autophagosome biogenesis (48). Given that *ptc1*Δ mutants show increased autophagic activity, we investigated whether these unusual septin architecture could be related to autophagosome formation. To this end, we performed a confocal fluorescence analysis of *ptc1*Δ cells coexpressing from their respective endogenous loci Cdc10-mCh and the autophagy protein Atg9 C-terminally tagged with the fluorophore mNeonGreen (mNG). We found that the punctate septin structures shown by daughters of multibudded *ptc1*Δ cells occasionally colocalized with Atg9 (Fig. 9), suggesting that they could be associated with the observed increase in autophagy.

### Slt2 localization is not altered in multibudded *ptc1*Δ cells.

**FIG 10:**
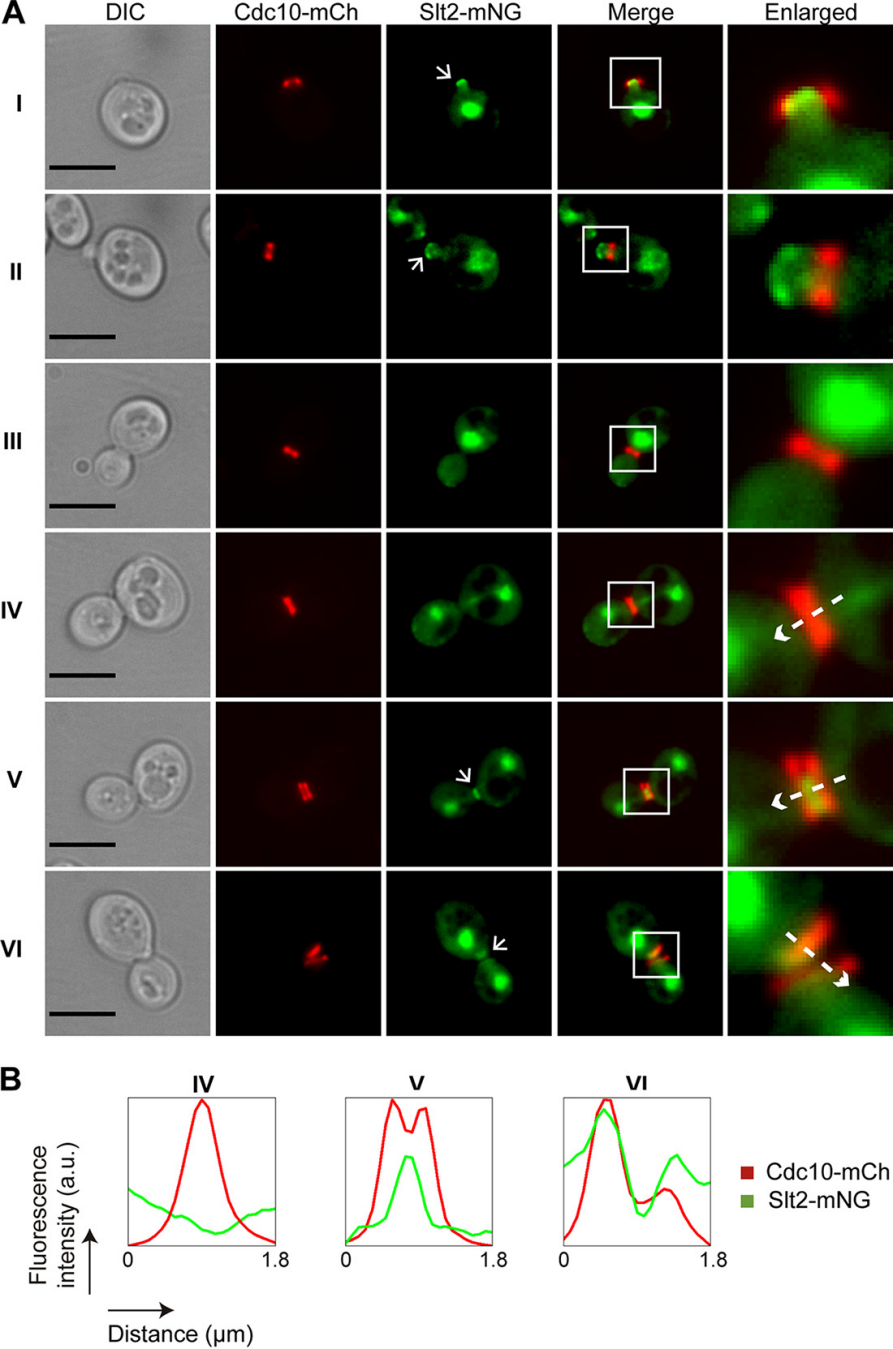
Localization of Slt2 and Cdc10 throughout the cell cycle of WT cells. (A) DIC and fluorescence microscopy images of exponentially growing cultures of strain YGGR17 (Slt2-mNG, CDC10-mCh). Bar = 5 μm. Arrows in the green channel (Slt2-mNG) point out the polarized localization of Slt2. (B) Histograms showing arbitrary units (a.u.) of fluorescence intensity versus distance represented by the dashed arrows in the enlarged images in panel A. These arrows indicate the direction in which the fluorescence intensity was measured, from the mother to the daughter cell.

**FIG 11:**
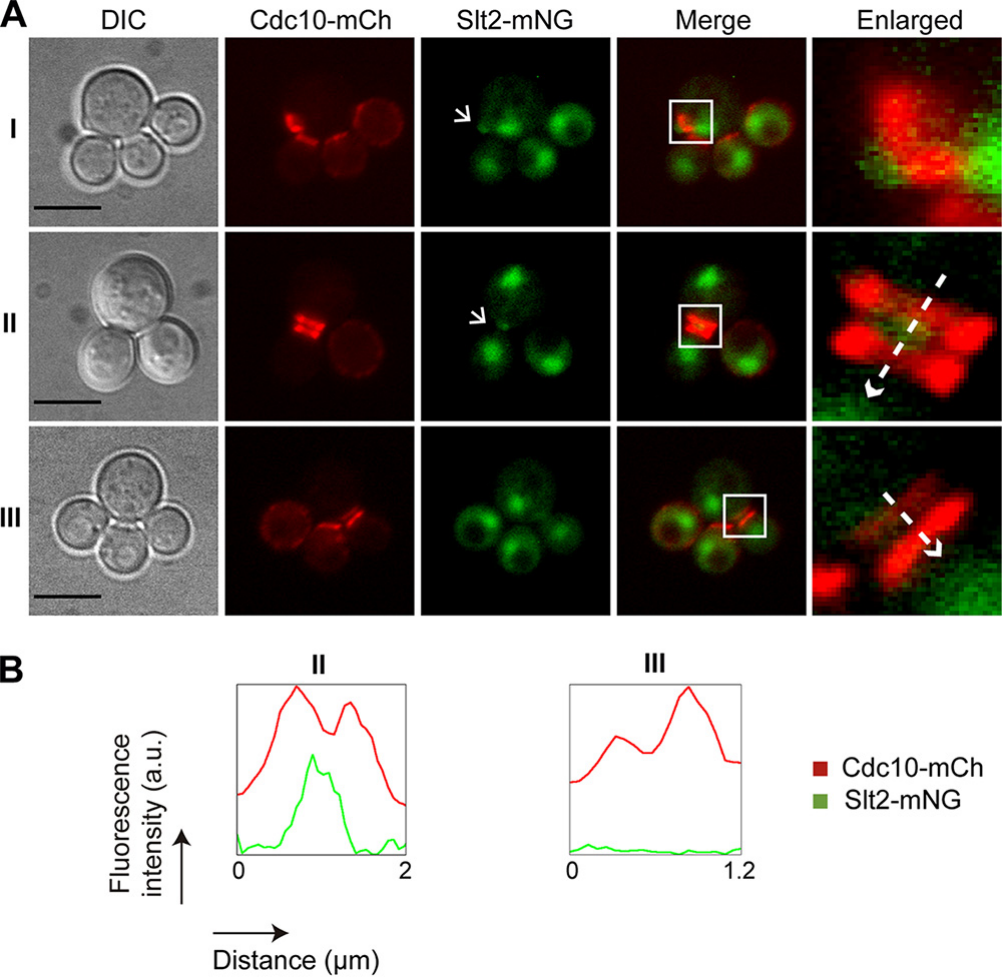
Localization of Slt2 and Cdc10 in multibudded *ptc1*Δ cells. (A) DIC and fluorescence microscopy images of exponentially growing cultures of strain YGGR18 (*ptc1*Δ Slt2-mNG, CDC10-mCh) incubated at 38°C for 6 h. Bar = 5 μm. Arrows in the green channel (Slt2-mNG) point out the polarized localization of Slt2. (B) Histograms showing arbitrary units (a.u.) of fluorescence intensity versus distance, represented by the dashed arrows in the enlarged images in panel A. These arrows indicate the direction in which the fluorescence intensity was measured, from the mother to the daughter cell.

Slt2 is predominantly localized in the nucleus at all stages of the cell cycle but also accumulates at sites of polarized growth, namely, the tip of small buds and the septa in late mitosis (49). Given that Slt2 regulates cell cycle progression through phosphorylation of different substrates (16, 18), we wanted to find out if Slt2 localization was altered in multibudded *ptc1*Δ cells. To better characterize Slt2 dynamics along the cell cycle, we coexpressed a C-terminally mNG-tagged version of Slt2 and septin CDC10-mCh, both from their endogenous loci. In WT cells, we observed that, in addition to its nuclear localization, Slt2 was found adjacent to the septin ring at the presumptive bud site at the beginning of the cell cycle (Fig. 10). Slt2 was also localized at the bud neck region, flanked by the septin double ring during cytokinesis until the separation of mother and daughter cells (Fig. 10). In cells lacking Ptc1 subjected to heat stress, Slt2 also accumulated in the nucleus and at sites of polarized growth (Fig. 11), as well as being localized between the double septin ring. However, contrary to what was observed for septins (Fig. 8), Slt2 was not abnormally retained on the daughter cell side in the septa of multibudded *ptc1*Δ cells (Fig. 11). These results indicate that Slt2 localization dynamics does not seem to be altered in cells lacking Ptc1.

### The RAM pathway is functional in multibudded *ptc1*Δ cells.

**FIG 12:**
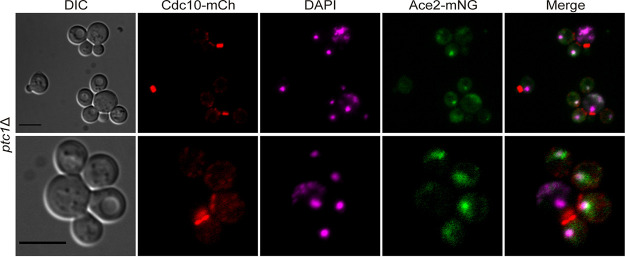
Localization of Ace2 and Cdc10 in multibudded *ptc1*Δ cells. DIC and fluorescence microscopy images of exponentially growing cultures of strain YGGR16 (*ptc1*Δ Ace2-mNG, Cdc10-mCh) incubated for 6 h at 38°C. Bar = 5 μm.

As mentioned above, the multibudding phenotype is a consequence of an asymmetric arrest of the cell cycle in daughter cells, which fail to complete cell separation and to start a new cell division cycle. Cell separation is promoted by the activation of the regulation of Ace2 and morphogenesis (RAM) pathway and the subsequent entry of the transcription factor Ace2 into the daughter cell nucleus (50). Hence, cells lacking RAM network function fail to carry out septum degradation, leading to a clumpy phenotype (51, 52). To assess whether the RAM network is functional in cells lacking Ptc1 subjected to heat stress, we studied the localization of Ace2. By fluorescence microscopy analysis of *ptc1*Δ cells stained with DAPI and coexpressing Cdc10-mCh and Ace2-mNG, we observed that Ace2 correctly localized to the daughter cell nucleus after septin collar splitting but remained in each daughter nucleus after successive mother cell division rounds (Fig. 12). This indicates that the cell separation defects exhibited by *ptc1*Δ mutants are not due to a failure of RAM pathway activation and suggests that events downstream of Ace2 entry into the nucleus underlie these alterations. Moreover, since during G_1_ progression, Ace2 is dephosphorylated and excluded from the nucleus (53), these results are consistent with the cell cycle arrest that occurs in *ptc1*Δ daughters but not in the mother cell.

### Cts1 does not localize to the septum of multibudded *ptc1*Δ cells.

**FIG 13:**
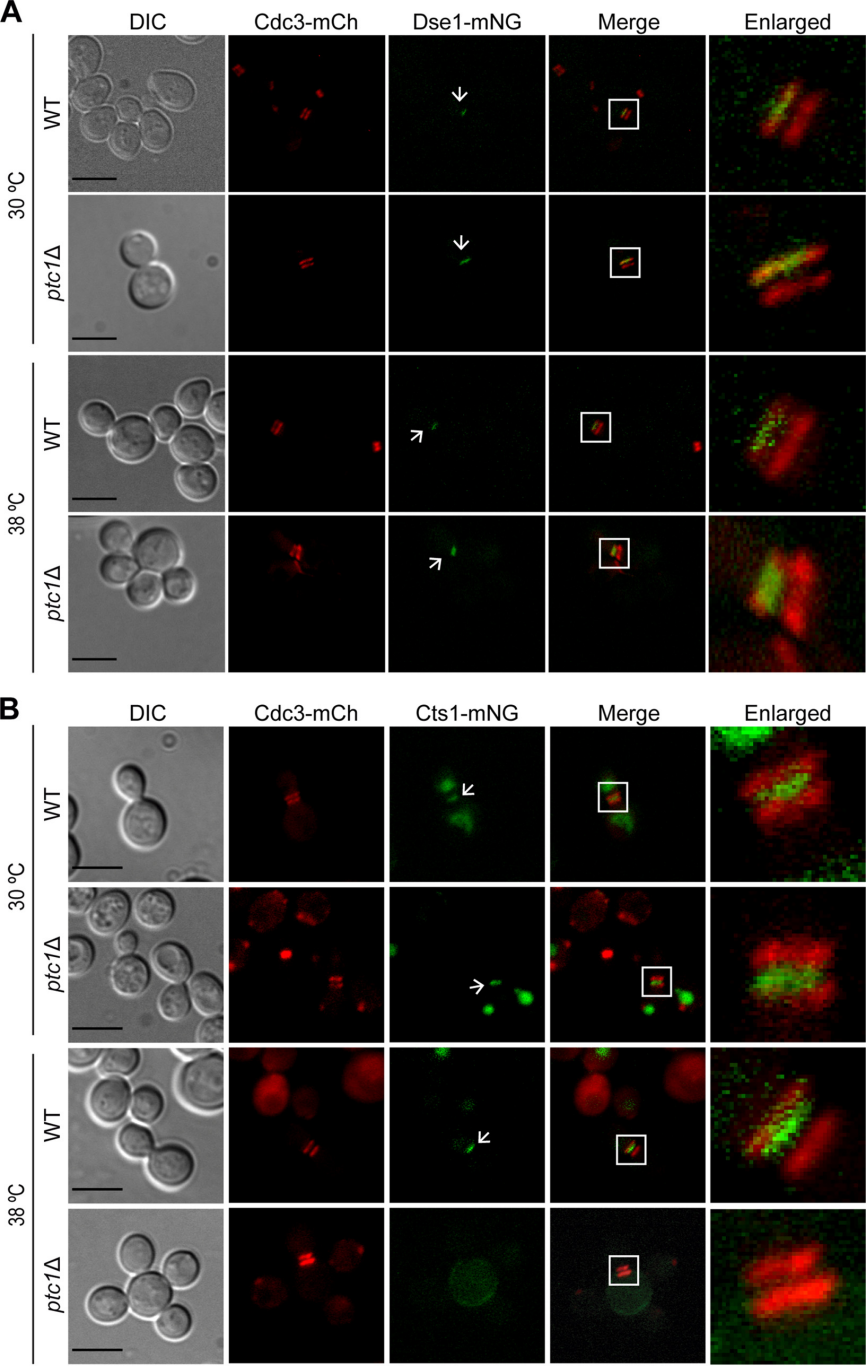
Localization of Dse1, Cts1 and Cdc3 in multibudded *ptc1*Δ cells. (A) Exponentially growing cells of YGGR54 (Dse1-mNG, Cdc3-mCh, WT) and YGGR55 (*ptc1*Δ Dse1-mNG, Cdc3-mCh) were incubated for 6 h at 30°C or 38°C and analyzed by DIC and fluorescence microscopy. (B) YGGR58 (Cts1-mNG, Cdc3-mCh, WT) and YGGR59 (*ptc1*Δ Cts1-mNG, Cdc3-mCh) cells were cultured and analyzed as for panel A. Bars = 5 μm.

Ace2 localization to the daughter cell nucleus induces the expression of several genes encoding the proteins mainly responsible for septum degradation, most notably the endochitinase Cts1, and others that are also part of the daughter-specific genetic program, such as Dse1 (6). Previous studies reported that both Dse1 and Cts1 localize at the daughter cell side of the septum (6, 54). With the aim of determining if the cell separation defects exhibited by *ptc1*Δ multibudded cells resulted from alterations on the localization of these proteins, we generated WT and *ptc1*Δ strains that coexpressed from their endogenous loci Cdc3-mCh and either Dse1-mNG or Cts1-mNG. We examined cells growing at 30°C or 38°C and observed Dse1-mNG localized at the daughter side of the septum in both WT and *ptc1*Δ strains at both temperatures (Fig. 13A). More specifically, in multibudded *ptc1*Δ cells, Dse1-mNG was found in the last-born daughter cell at cytokinesis (when the septin collar had split into two rings) (Fig. 13A). Regarding Cts1-mNG, in both WT and *ptc1*Δ cells growing at 30°C, Cts1-mNG also localized to the bud neck of budding yeast at cytokinesis, between the septin rings (Fig. 13B). Although Cts1-mNG fluorescence intensity decreased in the WT strain at 38°C, it was also found between the septin double ring (Fig. 13B). However, in 96% of multibudded *ptc1*Δ cells grown at 38°C, no Cts1-mNG was found in the septa with double septin rings (*n* = 70) (Fig. 13B), suggesting that cell separation defects in *ptc1*Δ cells could be due, at least in part, to a lack of proper endochitinase Cts1 localization at the septum.

## DISCUSSION

The regulation of yeast MAPK signaling pathways by Ptc1 was described a long time ago. However, whereas it is well established that Ptc1 negatively modulates the HOG and CWI MAPK cascades (21, 22), its role in the mating pathway is still controversial (25–27). Our results support the notion that Ptc1 favors pheromone signaling, since the absence of this protein phosphatase results in decreased levels of activated Fus3. Moreover, we observed a reduced signaling through Kss1, which unveils a potential role of Ptc1 in the regulation of this MAPK. Here, we also show that the *ptc1*Δ mutant displays a constitutive phosphorylation of Hog1 and Slt2 at 25°C, which is further evidence of the inhibitory function of Ptc1 in HOG and CWI pathways. However, only Slt2 is hyperphosphorylated at 38°C, suggesting that this MAPK is involved in the defects in growth and cell separation exhibited by cells lacking Ptc1 at high temperatures. This idea is supported by the finding that deletion of the MAPKK upstream Slt2, Mkk1, or the downstream transcription factor Rlm1 diminished these alterations (24, 28). However, deletion of *SLT2*, rather than alleviating, potentiates the *ptc1*Δ mutant phenotypic defects, leading to synthetic lethality in most genetic backgrounds (28), probably because the presence of Slt2 is essential to mediate the compensatory response against cell wall damage. By using an analog-sensitive version of Slt2 that allows for controlled inhibition of Slt2 kinase activity (38), we demonstrate here that the hyperactivity of Slt2 in cells lacking Ptc1 upon heat stress underlies the inability of daughter cells to separate after cytokinesis and initiate a new round of cell division.

Although loss of Ptc1 was associated with a transitory Slt2-dependent delay of mitochondrial inheritance (29), no alterations in mitochondrial function in *ptc1*Δ mutants had been previously described. Here, we show that the absence of Ptc1 in yeast subjected to thermal stress renders cells unable to inherit mitochondria and, consequently, mtDNA. Similar defects in mitochondrial and mtDNA inheritance in multibudded cells were previously observed in cytokinesis-defective *mdm10*Δ mutants (55, 56). In addition, we demonstrate that *ptc1*Δ mutants under heat stress exhibit higher mitochondrial membrane potential and increased ROS production and that both alterations are dependent on Slt2 kinase activity. We propose that, under CWI pathway-activating conditions, Slt2 hyperactivation renders cells unable to inherit mitochondria, contributing to the characteristic asymmetric cell cycle arrest that affects only the daughters of multibudded *ptc1*Δ cells. Hyperactive Slt2 could also be participating in a mitochondrion-dependent cell cycle arrest in *ptc1*Δ mutants through cyclin C, since it is known that cyclin C phosphorylation by Slt2 promotes mitochondrial fission (57, 58), and that a balance between mitochondrial fusion and fission is essential for regulating the cell cycle in yeast (59). In addition, the ability of the CWI pathway to stop the cell cycle progression when cells are exposed to different adverse conditions has been demonstrated by several studies (18). Remarkably, under TORC1-inhibiting conditions, the Slt2-dependent phosphorylation of the S-phase cyclin-dependent kinase (CDK) inhibitor Sic1 at threonine 173 contributes to its stabilization and consequently to G_1_ phase cell cycle arrest (60). Therefore, the TORC1 inhibition and excessive Slt2 kinase activity shown by stressed *ptc1*Δ cells could also contribute to the cell cycle arrest exhibited by daughter cells via phosphorylation of Sic1.

Both the accumulation of ROS and the inhibition of the TORC1-Sch9 pathway that we found in *ptc1*Δ cells could trigger autophagy, since both factors have been demonstrated to regulate this self-degradative process in yeast (45, 61, 62). Upon autophagy induction, septins could regulate autophagosome biogenesis, as previously suggested (48). Since peripheral septin structures are mainly localized in the daughters of multibudded cells, it seems that autophagy would be asymmetrically activated in daughter cells. Several studies have described a role of Slt2 in the control of autophagy, but this regulation differs mechanistically depending on the CWI pathway-activating agent. For example, upon rapamycin treatment, although phosphorylated, Slt2 is required neither for autophagy induction nor for TORC1 inhibition (63). However, Slt2 is required to trigger autophagy in response to DNA damage and ER stress, although this MAPK regulates only signaling via TORC1 in the latter case (32, 63). Hence, the role of Slt2 in regulating TORC1 inactivation and autophagy induction in *ptc1*Δ cells appears to be mechanistically similar to that observed under ER stress, which is in agreement with the reported alterations in the cortical ER (cER) inheritance displayed by this mutant (22, 29). Moreover, the results presented in this work, together with evidence that TORC1 is able to act negatively on Slt2 activity (64, 65), support the notion that Slt2 may exert regulatory feedback on TORC1 (66) to ensure robust and homeostatic cellular responses under changing environmental conditions.

Cells lacking Ptc1 display a characteristic vacuolar fragmentation phenotype (28, 67), which is substantially eliminated by deletion of *MKK1* (24). In addition, it is known that oxidative stress contributes to the inhibition of TORC1-Sch9 through vacuolar fragmentation and subsequent delocalization of Sch9 (68, 69). Thus, it is possible that the mechanism underlying Sch9 inhibition by Slt2 could result from a loss of vacuolar integrity caused by an increase in ROS. However, our results indicate that, while ROS are accumulated upon heat stress only in *ptc1*Δ mutants, TORC1-Sch9 inhibition is observed in both stressed and unstressed *ptc1*Δ cells. These data indicate that the inhibition of TORC1-Sch9 in *ptc1*Δ cells is not an effect of oxidative stress caused by Slt2 hyperactivation but a consequence of direct regulation of the TORC1 complex by this MAPK. In fact, Slt2 is already known to inhibit TORC2 through direct phosphorylation of the Avo2 subunit under CWI pathway-activating conditions (70).

Inhibition of TORC1, along with alterations in mitochondrial and cER inheritance, could contribute to the cell cycle arrest exhibited by the daughters of multibudded *ptc1*Δ cells, but why are these cells unable to dissociate from their mothers? In this work, we demonstrate that the cell separation defects shown by *ptc1*Δ mutants under heat stress are not due to a blockage in the RAM network, with Ace2 correctly localizing specifically to the daughter cell nucleus prior to cytokinesis. However, we observed that two proteins encoded by Ace2-regulated genes localized differently in the absence of Ptc1. While Dse1 was properly placed at the daughter side of the septum, as previously described (54), the endochitinase Cts1, which is the main enzyme responsible for cell separation, was not found in the septum of multibudded *ptc1*Δ cells. Since Ace2 accumulation in the daughter cell nucleus does not imply its association with target genes (71), we cannot rule out the possibility that Ace2 interacts with the Dse1 promoter but not with the Cts1 promoter in heat-stressed *ptc1*Δ cells. Nevertheless, it is more likely that the absence of Cts1 in its normal localization was due to a problem with reaching this site. Cts1 secretion depends on correct septation and subsequent degradation of Fir1, a protein localized at the cytokinesis site that is involved in a checkpoint-like mechanism termed the enforcement of cytokinesis order (ECO) pathway. Such a pathway ensures that cell separation occurs only when previous cytokinesis processes have been successfully completed. Although the signal that communicates the status of cytokinesis to Fir1 is still unknown, it has been hypothesized that the CWI pathway could sense the cell wall stress caused by septum defects (72). Considering that the activation of the CWI pathway promotes the expression of a subset of genes implicated in cell wall remodeling (73), hyperactivation of Slt2 in *ptc1*Δ mutants might lead to the synthesis of aberrant septa, preventing cell dissociation through Fir1 stabilization. Alternatively, given our observation that Slt2 localizes to the septum after septin collar splitting, septins or other substrates allocated there during cytokinesis could become hyperphosphorylated in *ptc1*Δ mutants, eventually causing the blockage of cell separation. In this sense, Fir1 as a target for Slt2 phosphorylation is a plausible possibility.

**FIG 14:**
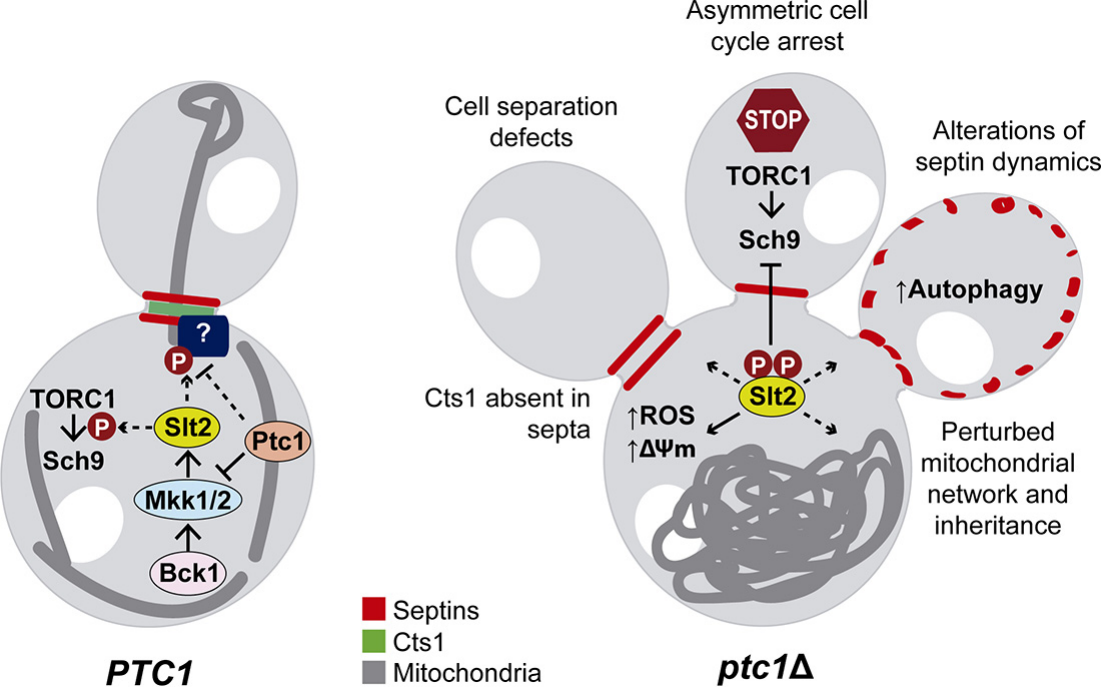
Model diagram in which the events related to the phenotypes described for *ptc1*Δ mutants are compared with wild-type cells.

Altogether, our results lead us to propose a model in which the combination of a permanent Slt2 hyperactivity and the absence of specific phosphatase activity provided by Ptc1 leads to an asymmetric cell cycle arrest by (i) perturbing mitochondrial network and inheritance, (ii) altering septin dynamics, (iii) inhibiting the TORC1-Sch9 signaling pathway, (iv) increasing autophagy, and (v) preventing the proper localization of Cts1 endochitinase at the septum and subsequent cell separation. We speculate that Slt2 is mediating these events through uncontrolled phosphorylation of key substrates that could also be dephosphorylation targets of Ptc1. Although the targets of Slt2 and Ptc1 involved in such processes remain to be uncovered, this work significantly expands the knowledge of the multiple CWI pathway functions (Fig. 14). The *ptc1*Δ mutant has proven to be an excellent platform to gain insight into the connections of signaling pathways with morphogenetic control circuits. Understanding all the details of this interface can be a challenging but alluring task, due to the conservation and importance of these regulatory mechanisms among eukaryotes.

## MATERIALS AND METHODS

### Yeast strains, plasmids, and culture conditions.

Yeast strains used in this study and their construction procedures can be found in Table 1. Plasmids are listed in Table 2, and oligonucleotide information is available upon request. Yeast cells were grown overnight at 30°C in yeast extract-Bacto peptone-dextrose (YPD) medium, or in a synthetic minimal medium containing 2% glucose when plasmid selection was required. Cultures were adjusted to an *A*_600_ of ~0.3 in YPD and incubated for 2 to 3 h to achieve the mid-exponential phase of growth. To perform solid growth assays, yeast cells were cultured to saturation overnight in liquid YPD and then adjusted to an *A*_600_ of ~0.5 in water. Tenfold serial dilutions of the cell suspensions were spotted onto YPD plates. When indicated, DTT (Sigma-Aldrich), CR (Merck), tunicamycin (Sigma-Aldrich), caffeine (Sigma-Aldrich), Zymolyase 100T (MP Biomedicals), and CFW (Sigma-Aldrich) were used as CWI pathway-activating compounds. For thermal stress, cells were incubated at 38°C.

**TABLE 1:** Strains used in this study

Strain	Genotype	Reference or source
BY4741	*MAT***a** *his3*Δ*1 leu2*Δ*0 met15*Δ*0 ura3*Δ*0*	76; Euroscarf
BY4741 *ptc1*Δ	BY4741 isogenic; *ptc1*Δ::*HIS3*	A. Sacristán-Reviriego
GFY-42	BY4741 isogenic; *cdc10*Δ::*CDC10*::mCherry::*ADH1(t)*::*SpHIS5*	77
Y3656	Δ*can1::MATaPr-HIS3* Δ*lyp1::MAT*α*Pr-LEU2* *his3*Δ*1* *leu2*Δ*0* *met15*Δ*0* *ura3*Δ*0*	78
Y02487	BY4741 isogenic; *mkk1*Δ::*kanMx4*	Euroscarf
Y02739	BY4741 isogenic; *rlm1*Δ::*kanMx4*	Euroscarf
Y04131	BY4741 isogenic; *swi6*Δ::*kanMx4*	Euroscarf
Y06109	BY4741 isogenic; *swi4*Δ::*kanMx4*	Euroscarf
YASS-1	Y3656 isogenic; *slt2* 323A→G, 324G→T (E108G)::*natMX6*	This study^*a*^
YGGR9	Y3656 isogenic; *cdc10*Δ::*CDC10*::mCherry::*ADH1(t)*::*SpHIS5*	This study^*b*^
YGGR10	YSTH14 isogenic; *cdc10*Δ::*CDC10*::mCherry::*ADH1(t)*::*SpHIS5*	This study^*b*^
YGGR12	YSTH14 isogenic; *ace2*Δ::*ACE2*::mNG::*ADH1(t)*::*HygR*	This study^*c*^
YGGR13	Isogenic Y3656; *slt2*Δ::*SLT2*::mNG::*ADH1(t)*::*HygR*	This study^*c*^
YGGR14	Isogenic YSTH14; *slt2*Δ::*SLT2*::mNG::*ADH1(t)*::*HygR*	This study^*c*^
YGGR16	YGGR12 isogenic; *cdc10*Δ::*CDC10*::mCherry::*ADH1(t)*::*SpHIS5*	This study^*b*^
YGGR17	Isogenic YGGR13; *cdc10*Δ::*CDC10*::mCherry::*ADH1(t)*::*SpHIS5*	This study^*b*^
YGGR18	Isogenic YGGR14; *cdc10*Δ::*CDC10*::mCherry::*ADH1(t)*::*SpHIS5*	This study^*b*^
YGGR19	YASS-1 isogenic; *ptc1*Δ::*kanMx4*	This study^*d*^
YGGR35	Y3656 isogenic; *swi4*Δ::*kanMx4*	This study^*e*^
YGGR38	Y3656 isogenic; *swi6*Δ::*kanMx4*	This study^*e*^
YGGR41	YSTH14 isogenic; *swi4*Δ::*kanMx4*	This study^*e*^
YGGR45	YGGR10 isogenic; *atg9*Δ::*ATG9*::mNG::*ADH1(t)*::*HygR*	This study^*c*^
YGGR47	YSTH14 isogenic; *swi6*Δ::*kanMx4*	This study^*e*^
YGGR52	IY3656 isogenic; *dse1*Δ::*DSE1*::mNG::*ADH1(t)*::*HygR*	This study^*c*^
YGGR53	YSTH14 isogenic; *dse1*Δ::*DSE1*::mNG::*ADH1(t)*::*HygR*	This study^*c*^
YGGR54	YGGR52 isogenic; *cdc3*-mCherry::*URA3*	This study^*f*^
YGGR55	YGGR53 isogenic; *cdc3*-mCherry::*URA3*	This study^*f*^
YGGR56	Y3656 isogenic; *cts1*Δ::*CTS1*::mNG::*ADH1(t)*::*HygR*	This study^*c*^
YGGR57	YSTH14 isogenic; *cts1*Δ::*CTS1*::mNG::*ADH1(t)*::*HygR*	This study^*c*^
YGGR58	YGGR56 isogenic; *cdc3*-mCherry::*URA3*	This study^*f*^
YGGR59	YGGR57 isogenic; *cdc3*-mCherry::*URA3*	This study^*f*^
YPL12	BY4741 isogenic; *rlm1*Δ::*kanMx4*; *ptc1*Δ::*HIS3*	L. Palacios
YPL14	BY4741 isogenic; *mkk1*Δ::*kanMx4*; *ptc1*Δ::*HIS3*	24
YSTH14	Y3656 isogenic; *ptc1*Δ::*natMX6*	79

a Constructed by cloning the SalI-BamHI fragment from pRS316-*slt2-as* (38) into SalI-BglII sites immediately preceding the nourseothricin *N*-acetyltransferase (*NAT*) gene (which confers resistance to nourseothricin; Jena Bioscience) of plasmid pFA6a-nat*MX6* (80). PCR amplifications bearing the whole *slt2*-as gene and the NAT marker were transformed into strain Y3656 in which the coding region of *SLT2* had been displaced by Kluyveromyces lactis *URA3*. Following selection on nourseothricin and 5-fluoroorotic acid, the mutants were sequenced to confirm the presence of the desired mutations.

b Strains carrying Cdc10 C-terminally fused in frame to the fluorophore mCherry (mCh) (81) were generated by integrating at the *CDC10* locus a DNA fragment containing *CDC10*::mCherry::*ADH1(t)*::*SpHIS5*, which was obtained by PCR from the genomic DNA of the GFY-42 strain.

c Strains expressing mNeonGreen (mNG) C-terminally tagged proteins (82) were generated by integrative transformation with a DNA fragment obtained by PCR amplification using the plasmid pAP67 as the template and containing the sequence mNG::*ADH1(t)*::*HygR* flanked by the regions located at the 5′ and 3′ ends of the termination codon of the gene of interest. Mutants were selected on 200 μg/mL hygromycin (Invitrogen), and the correct C-terminal fusion of the mNG tag in the genomic loci was checked by colony PCR.

d Constructed by gene replacement strategy (83), by an integrative transformation of the strain YASS-1 with a DNA fragment containing the kanamycin resistance marker flanked by noncoding 5′ and 3′ ends of *PTC1*, obtained by PCR amplification using the plasmid pFA6a-*kanMx4* as a template. Mutants were selected on 200 μg/mL G418 (Gibco, Life Technologies), and the correct gene replacement was checked by colony PCR.

e Constructed by transforming strains Y3656 and YSTH14 with the PCR products *swi4*Δ::*kanMx4* or *swi6*Δ::*kanMx4* obtained by PCR amplification from the genomic DNA of strains Y06109 and Y04131, respectively. Mutants were selected on 200 μg/mL G418, and the correct gene deletion was checked by colony PCR.

f Constructed by transforming BglII-digested YIp211-CDC3-mCherry (84) for its integration at the *CDC3* locus of the corresponding strains.

**TABLE 2:** Plasmids used in this study

Plasmid	Description	Source or reference
pAP67	pRS316; *prNIS*::*NIS*-mNG::*ADH1(t)*::*HygR*	J. W. Thorner
pFA6a-kanMx4	*kanMx4*	85
pFA6a-natMX6	*natMx6*	80
pOB08	pU326*-ilv6*-mCherry	O. A. Barbero
pRS316[*GFP-ATG8*] (pRS316 GFP-AUT7)	*CEN6 URA3 GFP-ATG8*	86
pRS316-*slt2-as*	*CEN6 URA3 slt2*-as	38
pRS416; *SCH9*-5HA	*CEN6 URA3 SCH9*-5HA	46
YIp211-*CDC3*-mCherry	Integrative, *URA3* mCherry (RFP)-*CDC3*	84

For Slt2-as inhibition assays, 2,3-DMB-PP1 (PP1 analog V) (Calbiochem) dissolved in dimethyl sulfoxide (DMSO) (PanReac Applichem ITW Reagents) was added to the medium before CWI pathway stress-inducing conditions.

For TORC1 pathway and autophagy studies, rapamycin (Cayman Chemical) was dissolved in DMSO and added to a final concentration of 0.2 μg/mL, and cycloheximide (Sigma-Aldrich) was dissolved in water and added to a final concentration of 25 μg/mL.

### Preparation of yeast extracts and immunoblotting analysis.

Cells were cultured as stated above, harvested by centrifugation, and stored at −80°C. Proteins were recollected by trichloroacetic acid precipitation and resuspended in 0.1 M Tris 5% SDS, as previously described (74). Once solubilized, samples were boiled for 5 min with 5× SDS-PAGE sample loading buffer, containing 1 M Tris-Cl (pH 6.8), 50% glycerol, 10% SDS, and 0.5% bromophenol blue (PanReac AppliChem ITW Reagents), and supplemented with 5% beta-mercaptoethanol (Sigma-Aldrich). For analysis of MAPK phosphorylation, GFP-Atg8 cleavage, and Sch9-5HA phosphorylation, cells extracts were resolved by SDS-PAGE using, respectively, 10%, 12%, and 8% gels containing acrylamide–bis-acrylamide (37.5:1). Gels were run at a constant voltage of 170 V until the bromophenol blue reached the bottom and transferred electrophoretically to 0.45-μm nitrocellulose blotting membranes (110 V for 70 min). The membranes were blocked by incubation in 5% nonfat dry milk diluted in 0.1% Tween 20 phosphate-buffered saline (PBS) at room temperature (RT) for 1 h, and then incubated in 1% nonfat dry milk 0.1% Tween 20 PBS containing a 1:1,000 dilution (unless stated otherwise) of the indicated primary antibody: mouse monoclonal anti-actin 69100 (MP Biomedicals), mouse monoclonal anti-GFP (JL8) (Clontech, TaKaRa), mouse monoclonal anti-HA (12CA5) (Roche), rabbit polyclonal anti-Hog1 y-215 (sc-9079) (Santa Cruz, Inc.), mouse monoclonal anti-Mpk1 (E9) (sc-133189) (Santa Cruz, Inc.), rabbit monoclonal anti-phospho-p-38 MAPK (Thr180/Tyr182) 3D7 (number 9215) (Cell Signaling) (1:500 dilution), or rabbit monoclonal anti-phospho-p44/42 MAPK (Erk1/2) (Thr202/Tyr204) (number 4370) (Cell Signaling). The membranes were then washed in 0.1% Tween 20–PBS (five times, for 5 min each) and incubated in the dark at room temperature (RT) for 1 h with a 1:5,000 dilution of an appropriate infrared dye-labeled secondary antibody: 800 CW goat anti-rabbit (926-68021), 800 CW goat anti-mouse (926-32210), 680LT goat anti-rabbit (926-68021), or 680LT goat anti-mouse (926-68020) (LI-COR Biosciences). After washes as described above, the resulting immune complexes were visualized using an Odyssey CLx infrared imaging system (LI-COR Biosciences).

### DAPI staining of nuclear and mtDNA.

The DAPI staining of nuclear and mtDNA was performed as follows. Yeasts growing in 1.5 mL of cell culture were harvested by brief centrifugation and washed once with deionized water. Then, cells were resuspended in PBS and incubated with 10 μg/mL or 5 μg/mL of DAPI (Thermo Fisher Scientific), for nuclear and mtDNA, respectively, in the dark at room temperature for 5 min and then rinsed three times with PBS. The stained cells were observed under fluorescence microscopy.

### DNA content analysis.

DNA content was analyzed with Sytox Green (Thermo Fisher Scientific) stain as previously described (34) with minor modifications. Briefly, ~1.5 × 10^7^ cells were harvested by centrifugation, washed once in 1 mL deionized water, resuspended in 4 mL of cold 70% ethanol for cell fixation and permeabilization, and kept overnight at 4°C. Fixed cells were collected by centrifugation, washed once in 1 mL deionized water, and incubated at 37°C for 2 h with 0.5 mL of RNase solution (2 mg/mL RNase A [Roche] in 50 mM Tris [pH 8], 15 mM NaCl; previously boiled and allowed to cool at RT). Then, cells were collected by centrifugation and incubated with 0.2 mL protease solution (5 mg/mL pepsin, 4.5 μg/mL HCl in deionized water) at 37°C for 15 min. Cells were collected by centrifugation and resuspended in 0.5 mL 50 mM Tris (pH 7.5). Finally, 100 μL of cell suspension was added to 1 mL of Sytox green solution (1 μM Sytox green in 50 mM Tris [pH 7.5]). Cells were analyzed by flow cytometry using a FACSCalibur instrument (Becton Dickinson) with an argon ion 15-mW laser tuned to 488 nm and an FL1 detector with a 530/30 band pass filter.

### PI staining and measurement of mitochondrial membrane potential.

Propidium iodide (PI) staining of S. cerevisiae cells was performed by incubating 1 mL of cell culture with 0.0005% PI (Sigma-Aldrich) at RT in the dark for 2 min. Stained cells were observed by fluorescence microscopy. A 1:10 dilution in PBS of the previous cell suspension was analyzed by flow cytometry using a FACScan instrument (Becton Dickinson) with excitation at 488 nm and the FL3 detector using the 650/LP long-pass filter.

Mitochondrial membrane potential was evaluated by flow cytometry in cells stained with rhodamine 123 (Rh 123). One milliliter of cell suspension was treated with Rh 123 (Sigma-Aldrich) at a final concentration of 5 μg/mL and aerobically incubated at 30°C in the dark for 30 min. Then, cells were stained with PI as stated above. Samples were diluted 1:10 in PBS and analyzed by flow cytometry using a FACScan instrument (Becton Dickinson) with excitation at 488 nm, the FL1 detector with a 530/30 band pass filter for Rh 123 analysis, and the FL3 detector with the 650/LP long-pass filter for PI analysis. PI-positive cells were excluded from the analysis of Rh 123-derived fluorescence.

### ROS detection.

ROS production was quantified by flow cytometry using dihydroethidium (DHE) staining. Cell suspensions were incubated with 2.5 μg/mL DHE at 30°C for 5 min in the dark. Samples were diluted 1:10 in PBS and analyzed by flow cytometry using a FACScan (Becton Dickinson) with excitation at 488 nm and the FL3 detector using the 650/LP long-pass filter.

Flow cytometry data analysis was performed with FlowJo v10 software (BD Biosciences).

### Fluorescence microscopy.

For fluorescence microscopy analysis, cells stained as stated above, or cells expressing proteins tagged with mCherry (mCh) or mNeonGreen (mNG) were examined with an Eclipse TE2000U inverted microscope (Nikon) equipped with a 100× oil immersion objective, and a super-high-pressure mercury lamp for power supply. Digital images were captured using an Orca C4742-95-12ER charge-coupled-device camera (Hamamatsu Photonics) and processed by HCImage software (Hamamatsu). Samples used to assess localization of Slt2 and Cdc10 throughout the cell cycle were imaged using an upright epifluorescence microscope (Olympus; model BH-2) equipped with a 100× oil immersion lens (Olympus) and a SOLA light source (Lumencore). Images were acquired with a charge-coupled device camera (CoolSNAP MYO; Photometrics) and processed by Micro-Manager software. Samples used to assess inheritance of mitochondria and mtDNA were imaged using an inverted epifluorescence Leica DMi8 microscope equipped with an Apochromat 100× oil immersion objective (Leica Microsystems, Wetzlar, Germany) and a CoolLED pE300 light source (CoolLED Ltd., Andover, UK). Images were acquired with a Leica K8 charge-coupled device camera and processed by LAS X 3.8.0 software (Leica Microsystems, Wetzlar, Germany).

For confocal laser microscopy, cells expressing mCh- and mNG-tagged fluorescent proteins were treated as previously described (75), with minor modifications: yeasts growing in 1.5 mL of cell culture were collected by centrifugation and fixed with 4% paraformaldehyde–3.4% sucrose at RT for 15 min. Cells were washed once in PBS and resuspended in 200 μL of this buffer. For coverslip coating, 5 μL of 1 mg/mL concanavalin A type IV-S (Sigma-Aldrich) was spread over standard coverslips, 18 by 18 mm and 0.13 to 0.16 mm thick (Labbox), with a pipet tip and let dry at RT. For yeast immobilization, 7 μL of fixed cell suspension was added to the coated coverslips and let dry at RT. Then, 20 μL ProLong Glass antifade mountant (Thermo Fisher)–glycerol (1:1) was applied to the coverslip, which was then lowered on a microscope slide and allowed to cure overnight at RT. Mounted samples were sealed with nail polish and examined with a LSCM Leica SP8 (Leica) laser scanning confocal microscope. Confocal images were processed with Leica Application Suite X (LAS X) software.

Microscope images were analyzed using ImageJ (National Institutes of Health).
